# Allicin suppresses preharvest soft rot associated with a *Diaporthe* sp. and enhances defense responses in ‘Ancui’ hardy kiwi

**DOI:** 10.3389/fpls.2026.1938269

**Published:** 2026-09-09

**Authors:** Zihuan Hou, Lixin Wang, Jiaying Huang, Shaofeng Jia, Yali Zhang, Yanan Li, Jinying Li, Lin Wu, Ying Wang

**Affiliations:** 1 College of Horticulture, Jilin Agricultural University, Changchun, China; 2 Institute of Pomology, Jilin Academy of Agricultural Sciences (Northeast Agricultural Research Center of China), Gongzhuling, China

**Keywords:** *Actinidia arguta*, allicin, defense enzymes, *Diaporthe* sp., oxidative damage, preharvest soft rot, sustainable disease management

## Abstract

Preharvest soft rot poses a threat to the commercial development of ‘Ancui’ hardy kiwi (*Actinidia arguta*), a newly developed cultivar with increasing cultivation potential in the cold northern regions of China. However, the fungus associated with this disease and environmentally friendly strategies for its management remain insufficiently understood. In this study, a fungal isolate was recovered from symptomatic fruits and characterized through morphological observation, internal transcribed spacer (ITS) sequence analysis, phylogenetic analysis, and pathogenicity tests. Based on the combined evidence, the fungus was assigned to the genus Diaporthe and conservatively designated as *Diaporthe* sp. isolate B4. A commercial 6% allicin emulsifiable concentrate exhibited concentration-dependent antifungal activity against the isolate *in vitro*, with an EC_50_ of 326.90 mg/L on a formulation-concentration basis. In a field trial, two applications of allicin at 800–1,000 mg/L provided the highest disease control efficacy, reaching approximately 82%. Allicin treatment also reduced superoxide anion production and malondialdehyde accumulation while increasing the activities of superoxide dismutase, peroxidase, catalase, and phenylalanine ammonia-lyase in fruit tissues. These findings suggest that the suppression of preharvest soft rot by allicin may involve both direct antifungal activity and enhanced defense-related responses in hardy kiwi fruit. Allicin therefore represents a promising botanical option for the sustainable management of preharvest soft rot in ‘Ancui’ hardy kiwi.

## 1 Introduction

Hardy kiwi (*Actinidia arguta* (Siebold & Zucc.) Planch. ex Miq.) is a perennial fruit crop belonging to the family Actinidiaceae and is valued for its distinctive flavor, nutritional quality, and strong cold tolerance. Its cultivation has expanded in northern China, particularly in cold mountainous regions where the crop is well adapted (Wang, 2023; Sun et al., 2025). ‘Ancui’ is a newly bred hardy kiwi cultivar developed by the College of Horticulture, Jilin Agricultural University, in collaboration with the *A. arguta* Science and Technology Courtyard in Hunjiang, Jilin Province. The cultivar has shown promising cultivation potential in cold northern production areas. However, the expansion of planted area, increasing orchard density, and prolonged cultivation have been accompanied by more frequent disease outbreaks, which increasingly threaten fruit yield, fruit quality, and the sustainable development of hardy kiwi production.

Hardy kiwi production is affected by several fungal and bacterial diseases, including black spot caused by *Alternaria* spp., brown spot caused by *Corynespora cassiicola*, anthracnose caused by *Colletotrichum gloeosporioides*, soft rot associated with *Diaporthe* spp., and bacterial canker caused by *Pseudomonas syringae* pv. *actinidiae* (Zhao et al., 2013; Lyu, 2024; Pereira et al., 2021). Related fungal fruit diseases, including black spot, anthracnose, bitter rot, and postharvest fruit rot, have also been reported in other horticultural crops, such as persimmon, pear, apple, and strawberry, as well as in kiwifruit (Kobiler et al., 2011; Wu et al., 2010; Zhang et al., 2009; Ren, 2007; Yang et al., 2018). Among these diseases, fruit soft rot is particularly important because it can develop during both preharvest and postharvest stages. In orchards in Hunjiang District, Jilin Province, typical symptoms frequently appear during late fruit development and include water-soaked lesions, tissue softening, and rapid fruit decay. Previous field observations showed that water-soaked softening around the fruit stalk occurred as early as late July, with disease incidence ranging from 0.9% to 5.7% (Qiao et al., 2024). *Botryosphaeria dothidea* and *Diaporthe* spp. have also been reported as important fungi associated with kiwifruit decay (Li et al., 2017; Wang et al., 2021), and favorable temperature and humidity conditions can accelerate tissue collapse (Shi et al., 2020). However, the fungal pathogen associated with preharvest soft rot of the newly bred cultivar ‘Ancui’ in cold northern production areas has not been adequately characterized, limiting the development of targeted disease management strategies.

Current management of kiwifruit fruit diseases relies largely on chemical fungicides and cultural practices. Although synthetic fungicides can provide effective disease suppression, their repeated and long-term use may contribute to fungicide resistance, pesticide residues, and environmental contamination, raising concerns regarding ecological and food safety (Ge et al., 2020; Fontana et al., 2021). Consequently, increasing attention has been directed toward environmentally compatible alternatives, including botanical pesticides, microbial metabolites, essential oils, and other biorational antifungal products (Lyu et al., 2017; Han et al., 2018; Xu et al., 2024; Huang et al., 2024). Among these alternatives, allicin, a sulfur-containing bioactive compound derived primarily from garlic (*Allium sativum*), has attracted interest because of its broad-spectrum antimicrobial activity against numerous plant-pathogenic fungi and bacteria (Kim et al., 2012; Hayat et al., 2016; Zhang et al., 2020; Tan and Tao, 2024). Its natural origin, biodegradability, and antimicrobial potential suggest that allicin may serve as a promising botanical agent for low-residue disease management in fruit crops. The potential value of allicin for fruit disease management may extend beyond its direct antifungal activity. Previous biochemical studies have shown that allicin can interact with thiol-containing proteins and readily permeate phospholipid membranes, properties that may contribute to its biological activity (Rabinkov et al., 1998; Miron et al., 2000). As a reactive sulfur-containing compound, allicin may influence redox homeostasis in host tissues and thereby affect fruit responses to pathogen infection. At appropriate concentrations, allicin may alleviate pathogen-induced oxidative damage and stimulate defense-related responses, whereas excessive exposure could disrupt cellular redox balance, aggravate membrane lipid peroxidation, or impair antioxidant enzyme activity. Therefore, evaluating changes in oxidative stress indicators and defense-related enzymes is essential for determining whether allicin-mediated disease suppression is associated with improved physiological resistance in hardy kiwi fruit. However, systematic information on the direct antifungal activity, field efficacy, and host physiological effects of allicin against preharvest soft rot of hardy kiwi remains limited.

In this study, symptomatic ‘Ancui’ hardy kiwi fruits were collected from orchards in Hunjiang District, Jilin Province, China, and the fungal isolate associated with preharvest soft rot was characterized through morphological observation, ITS sequence analysis, phylogenetic analysis, and pathogenicity tests. The biological characteristics of the isolate were examined, and the antifungal activity of a commercial 6% allicin emulsifiable concentrate was evaluated using *in vitro* dose–response assays and a field trial. In addition, superoxide anion production, malondialdehyde accumulation, and the activities of superoxide dismutase, peroxidase, catalase, and phenylalanine ammonia-lyase were measured to determine whether disease suppression by allicin was associated with reduced oxidative damage and enhanced defense-related responses. This study aimed to characterize the fungal pathogen associated with preharvest soft rot of ‘Ancui’ hardy kiwi and to evaluate the potential of allicin as a botanical option for sustainable disease management.

## 2 Materials and methods

### 2.1 Materials

‘Ancui’ hardy kiwi (*Actinidia arguta*) fruits were collected from the *A. arguta* Science and Technology Courtyard in Hunjiang District, Jilin Province, China. Uniform, plump, and intact fruits without visible mechanical damage or disease symptoms were selected at approximately 80% commercial maturity. The soluble solids content of the selected fruits ranged from 6.0% to 8.0%, as measured using a digital refractometer. After harvest, the fruits were immediately transported to the laboratory and stored at 4 °C until use. Potato dextrose agar (PDA) was used as the basal culture medium. The botanical fungicide tested in this study was a commercial 6% allicin emulsifiable concentrate (EC; 6 ag active ingredient per 100 mL formulation; Shaanxi Ciyuan Biotechnology Co., Ltd., China). Unless otherwise stated, all allicin concentrations reported in this study refer to the concentrations of the commercial 6% allicin EC formulation rather than the concentrations of the active ingredient. Other reagents included 70% methanol, 75% ethanol, 1% sodium hypochlorite, an Ezup Column Fungal Genomic DNA Extraction Kit (Sangon Biotech, Shanghai, China), and universal internal transcribed spacer primers for fungal identification.

### 2.2 Pathogen isolation and identification

#### 2.2.1 Isolation and purification of the pathogen

Pathogen isolation was performed using a tissue isolation method with minor modifications (Suwannarach et al., 2025). A total of 50 symptomatic fruits were collected from three independent orchards, from which five representative fruits exhibiting typical water-soaked soft rot symptoms were selected for fungal isolation. The fruits were rinsed with sterile distilled water for 5 min, and small tissue blocks were excised from the margins between diseased and healthy tissues under aseptic conditions. The tissue blocks were surface-disinfested in 75% ethanol for 1 min, rinsed once with sterile distilled water, immersed in 1% sodium hypochlorite for 2 min, and subsequently rinsed three times with sterile distilled water for 30 s each. After air-drying on sterile filter paper, tissue blocks of approximately 5 mm × 5 mm were placed on PDA plates and incubated at 28 °C for 72 ah. Uninoculated PDA plates were incubated simultaneously as sterility controls. Colonies with distinct morphological characteristics were selected and purified through three successive rounds of single-spore isolation. The purified isolates were maintained on PDA slants at 4 °C for subsequent experiments. The isolation and purification procedures included three biological replicates.

#### 2.2.2 Morphological identification

Purified isolates were inoculated onto PDA plates and incubated at 25 °C for 7 ad. Colony morphology, color, texture, and radial growth were recorded during incubation. For microscopic examination, mycelium was randomly collected from the colony margins, and fragments measuring approximately 5 mm × 5 mm were excised and mounted on microscope slides. Hyphal morphology, septation, conidiophores, and conidia were observed and photographed using a light microscope according to previously described methods (Li et al., 2021). Three biological replicates were included for morphological characterization.

#### 2.2.3 Molecular identification

Genomic DNA was extracted from purified fungal mycelia using an Ezup Column Fungal Genomic DNA Extraction Kit according to the manufacturer’s instructions. Three parallel DNA samples were prepared for polymerase chain reaction (PCR) verification. DNA integrity was assessed by electrophoresis on a 1.0% agarose gel, and DNA purity was determined using a microvolume UV spectrophotometer. DNA samples with an A260/A280 ratio of 1.8–2.0 were used for subsequent amplification.

The internal transcribed spacer (ITS) region was amplified using the universal primers ITS1 (5′-TCCGTAGGTGAACCTGCGG-3′) and ITS4 (5′-TCCTCCGCTTATTGATATGC-3′). Each 25 μL PCR reaction contained 12.5 μL of 2× Taq PCR Master Mix, 1 μL of each primer, 2 μL of DNA template, and 8.5 μL of sterile ddH_2_O. The amplification program consisted of an initial denaturation at 94 °C for 4 min; 35 cycles of denaturation at 94 °C for 30 s, annealing at 55 °C for 30 s, and extension at 72 °C for 1 min; followed by a final extension at 72 °C for 10 min. The PCR products were examined by electrophoresis on a 1.0% agarose gel and submitted to Changchun Kumei Biotechnology Co., Ltd. (Changchun, China) for sequencing.

The resulting ITS sequence was compared with sequences available in the National Center for Biotechnology Information database using the Basic Local Alignment Search Tool. Multiple sequence alignment was performed using ClustalX 2.1 (Larkin et al., 2007). A phylogenetic tree was constructed in MEGA 11 using the neighbor-joining method with 1,000 bootstrap replicates (Tamura et al., 2021). Reference sequences were selected according to their taxonomic reliability, sequence similarity, and relevance to *Diaporthe* species or fruit-tree diseases. The preliminary taxonomic placement of the isolate was inferred from sequence similarity, phylogenetic clustering, and morphological characteristics. The 548-bp ITS sequence of *Diaporthe* sp. isolate B4 was deposited in GenBank under accession number PZ789839.

#### 2.2.4 Pathogenicity verification

The pathogenicity of the isolate was assessed by wound inoculation under field conditions. Thirty healthy ‘Ancui’ fruits were randomly assigned to an inoculation group and a control group, with 15 fruits per group. A 5-mm-diameter mycelial plug was excised from the actively growing margin of a 7-day-old colony and placed on a shallow artificial wound approximately 2 mm deep at the equatorial region of each fruit. Control fruits were inoculated with sterile PDA plugs of the same size. Moist cotton and perforated breathable bags were placed around the inoculation sites to maintain approximately 90% relative humidity for 48 ah. All fruits were subsequently maintained under natural field conditions at an average temperature of approximately 25 °C.

Symptom development was monitored and recorded daily for 7 ad, and disease severity was evaluated according to the proportion of the lesion area relative to the total fruit surface area. At 7 ad post-inoculation, the fungus was re-isolated from tissues collected at the margins between diseased and healthy areas. The colony morphology and microscopic characteristics of the re-isolated strains were compared with those of the original isolate to fulfill Koch’s postulates.

### 2.3 Biological characteristics of the pathogen

The effects of culture medium, nitrogen source, carbon source, pH, temperature, and light conditions on mycelial growth were evaluated according to Du et al. (2021). Unless otherwise stated, PDA was used as the basal medium. Mycelial plugs measuring 5 mm in diameter were excised from the actively growing margins of 7-day-old colonies and placed in the centers of the corresponding media. The cultures were incubated under the specified conditions for 7 ad. Colony diameter was measured daily along two perpendicular axes, and the mean value was used to calculate the average daily mycelial growth rate.

#### 2.3.1 Effects of culture media on mycelial growth

To determine the effects of culture medium on mycelial growth, six media were evaluated: potato carrot agar (PCA), Czapek’s medium, Rose Bengal chloramphenicol agar (RBC), water agar (WA), nutrient agar (NA), and potato sucrose agar (PSA). Before inoculation, all media were adjusted to pH 7.0 and autoclaved at 121 °C for 20 min.

#### 2.3.2 Effects of nitrogen sources on mycelial growth

To determine the effects of nitrogen source on mycelial growth, Czapek’s medium was used as the basal medium. Sodium nitrate (NaNO_3_), originally present at 2 g/L, was separately replaced with beef extract, peptone, yeast extract, ammonium sulfate, ammonium nitrate, or urea on an equivalent nitrogen basis. Czapek’s medium without an added nitrogen source was used as the nitrogen-free control.

#### 2.3.3 Effects of carbon sources on mycelial growth

To determine the effects of carbon source on mycelial growth, Czapek’s medium containing 30 g/L sucrose was used as the basal medium. Sucrose was separately replaced with glucose, sorbitol, mannitol, lactose, or soluble starch on an equivalent carbon basis. Czapek’s medium without an added carbon source was used as the carbon-free control.

#### 2.3.4 Effects of light conditions on mycelial growth

To determine the effects of light conditions on mycelial growth, three light regimes were evaluated: continuous light, continuous darkness, and a 12 ah light/12 ah dark photoperiod. All cultures were incubated at 25 °C for 7 ad.

#### 2.3.5 Effects of temperature on mycelial growth

To determine the effects of temperature on mycelial growth, mycelial plugs were inoculated onto PDA plates and incubated at 5, 10, 15, 20, 25, 30, 35, or 40 °C for 7 ad.

#### 2.3.6 Effects of pH on mycelial growth

To determine the effects of pH on mycelial growth, PDA media were adjusted to pH 4, 5, 6, 7, 8, 9, 10, or 11 using hydrochloric acid or sodium hydroxide before sterilization. After inoculation, the cultures were incubated at 25 °C for 7 ad.

### 2.4 Evaluation of allicin against soft rot

#### 2.4.1 *In vitro* inhibition and toxicity regression analysis

The *in vitro* antifungal activity of a commercial 6% allicin emulsifiable concentrate (EC) against the *Diaporthe* isolate was evaluated by measuring mycelial growth on allicin-amended PDA. The formulation was diluted with sterile distilled water to prepare working solutions, which were incorporated into autoclaved PDA cooled to approximately 40 °C at a volume ratio of 1:100 to obtain final formulation concentrations of 50, 200, 400, 600, 800, 1,000, and 1,200 mg/L. PDA supplemented with an equivalent volume of sterile distilled water was used as the blank control.

A 5-mm-diameter mycelial plug was excised from the actively growing margin of a 7-day-old colony and placed in the center of each plate. All plates were incubated at 28 °C for 7 ad, with three replicate plates per treatment. Colony diameter was measured after incubation, and the mycelial growth inhibition rate was calculated using the following Equation 1:

(1) $$\text{Inhibition rate}\left(\%\right)=\frac{\text{colony diameter of control}-\text{colony diameter of treatment}}{\text{colony diameter of control}}\times 100$$

The concentration–response relationship was subsequently analyzed using probit regression. The mean inhibition rate at each concentration was converted to a probit value, and the log_10_-transformed allicin concentration was used as the independent variable. The probit regression equation, median effective concentration (EC_50_), effective concentration causing 90% inhibition (EC_90_), coefficient of determination (R^2^), and corresponding 95% confidence intervals were estimated from the fitted model.

#### 2.4.2 Field control efficacy of allicin

Field efficacy was evaluated in an orchard in Hunjiang District, Jilin Province, China (41°43′ N, 126°25′ E), from August to September 2025. Five-year-old ‘Ancui’ hardy kiwi trees with uniform growth were selected for the trial. During the experimental period, the mean temperature ranged from 22 to 28 °C, and the relative humidity ranged from 65% to 85%. Disease development occurred under natural infection conditions, and no other fungicides were applied during the trial. Eight treatments were established, comprising seven allicin concentrations (50, 200, 400, 600, 800, 1,000, and 1,200 mg/L) and a sterile-water control. The experiment was arranged in a randomized block design with three replicate plots per treatment. Each plot covered 66.7 m^2^ and contained 12 uniformly growing trees. Within each plot, five trees were selected using a diagonal five-point sampling method. Twenty fruits of uniform size and without visible mechanical damage were randomly selected and labeled on each tree, resulting in a total of 100 fruits per plot for disease assessment.

The first application was performed on 23 August 2025 using a hand-held sprayer. A second application was conducted 7 ad later. During each application, both the upper and lower leaf surfaces were wetted first, followed by spraying of the fruit surface and fruit stalk base until fine droplets uniformly covered the fruit surface without runoff. Control plots were sprayed with an equivalent volume of sterile water using the same procedure. Disease assessments were conducted before the first application and 7 ad after each application. The same 100 labeled fruits in each plot were assessed at each evaluation time. Disease severity was assessed using a 0–9 grading scale according to Zhu (2023): grade 0, no visible lesions; grade 1, lesions covering >0–10% of the total fruit surface area; grade 3, 11–25%; grade 5, 26–50%; grade 7, 51–75%; and grade 9, >75%. The disease index, disease index increment, and field control efficacy were calculated using Equations 2–4:

(2) $$\text{Disease index}=\frac{\sum \left(\text{disease grade}\times \text{number of fruits at the corresponding grade}\right)}{\text{total number of fruits}\times \text{highest disease grade}}\times 100$$

(3) Disease index increment=disease index after application−disease index before application.

(4) $$\text{Control efficacy}\left(\%\right)=\frac{\text{disease index increment of control}-\text{disease index increment of treatment}}{\text{disease index increment of control}}\times 100$$

### 2.5 Determination of oxidative stress indicators and defense-related enzyme activities

Fruit samples were collected 7 ad after the second allicin application. For each treatment, 10 fruits of uniform size were randomly collected from each replicate plot. Pulp tissues from the equatorial region of the fruits were pooled, immediately frozen in liquid nitrogen, and stored at −80 °C until analysis. Approximately 0.5 ag of frozen pulp tissue was homogenized on ice with 5 mL of pre-cooled 0.1 mol/L phosphate buffer (pH 7.0). The homogenate was centrifuged at 12,000 × g for 20 min at 4 °C, and the resulting supernatant was collected as the crude enzyme extract.

The MDA content and the activities of SOD, POD, and CAT were determined using the thiobarbituric acid, nitroblue tetrazolium reduction, guaiacol, and ultraviolet absorption methods, respectively, according to Yang et al. (2021). PAL activity was determined using the borate buffer method. The O_2_•^−^ production rate was determined using a spectrophotometric method. All measurements were expressed on a fresh-weight basis. The units were defined as follows: O_2_•^−^ production rate, μmol min^−1^ g^−1^ FW; MDA content, μmol g^−1^ FW; POD activity, U g^−1^ FW min^−1^; SOD activity, U g^−1^ FW; PAL activity, U g^−1^ FW h^−1^; and CAT activity, U g^−1^ FW min^−1^. All physiological indicators were measured at the same sampling time, with three biological replicates per treatment.

### 2.6 Statistical analysis

Data are presented as the mean ± standard deviation (SD). Differences among treatments were analyzed using one-way analysis of variance (ANOVA), followed by Duncan’s multiple range test at P< 0.05. Probit regression was used to analyze the concentration–response relationship between allicin concentration and mycelial growth inhibition. The probit regression equation, median effective concentration (EC_50_), effective concentration causing 90% inhibition (EC_90_), coefficient of determination (R^2^), and corresponding 95% confidence intervals were calculated from the fitted model.

Spearman rank correlation analysis was performed among allicin concentration, field control efficacy, disease index, O_2_•^−^ production rate, MDA content, and SOD, POD, CAT, and PAL activities. Because field control efficacy was calculated relative to the sterile-water control, the control treatment was excluded from the correlation analysis. The analysis included seven allicin concentrations with three biological replicates per concentration (n = 21). Statistical analyses were performed using SPSS Statistics 26.0. Figures and heatmaps were prepared using OriginPro 2024 and GraphPad Prism 10.0.

## 3 Results

### 3.1 Natural symptoms and pathogenicity of isolate B4

**Figure 1:**
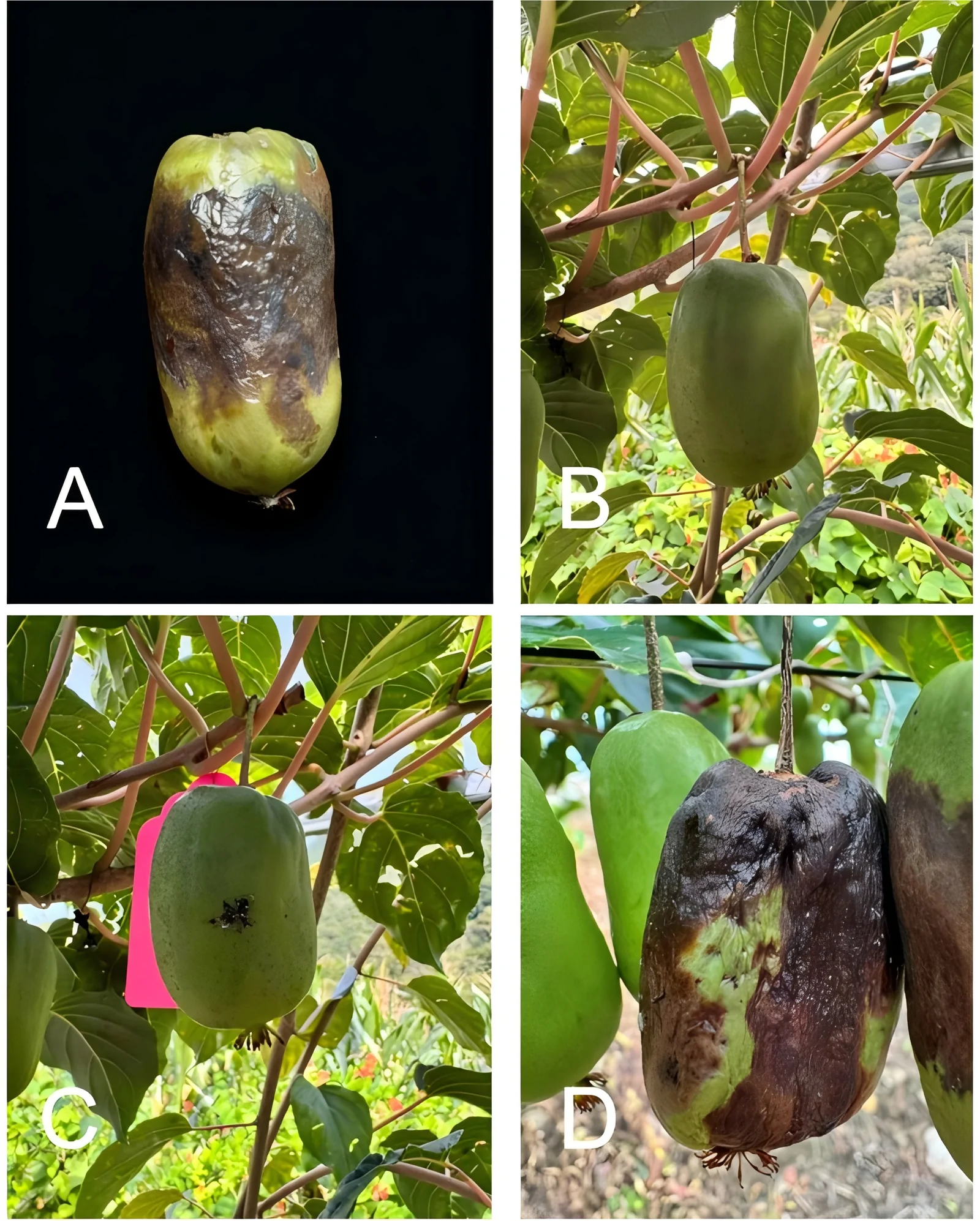
Natural soft rot symptoms of ‘Ancui’ hardy kiwi and disease progression at 7 ad post-inoculation with *Diaporthe* sp. isolate B4. **(A)** Naturally diseased fruit. **(B)** Healthy fruit before inoculation. **(C)** Early lesions after inoculation. **(D)** Advanced soft rot symptoms after inoculation.

Naturally infected ‘Ancui’ hardy kiwi fruits exhibited typical soft rot symptoms, characterized by water-soaked lesions, tissue softening, and dark brown decayed areas on the fruit surface. Following wound inoculation with isolate B4, brown necrotic lesions initially developed around the inoculation sites and subsequently expanded into water-soaked, softened areas resembling those observed on naturally infected fruits. In contrast, fruits inoculated with sterile PDA plugs remained symptomless throughout the 7-d observation period. The fungus was successfully re-isolated from the symptomatic tissues of inoculated fruits, and its colony morphology and microscopic characteristics were consistent with those of the original isolate. These results fulfilled Koch’s postulates and demonstrated that isolate B4 was pathogenic to ‘Ancui’ hardy kiwi fruit (**Figure 1**).

### 3.2 Morphological characteristics of isolate B4

**Figure 2:**
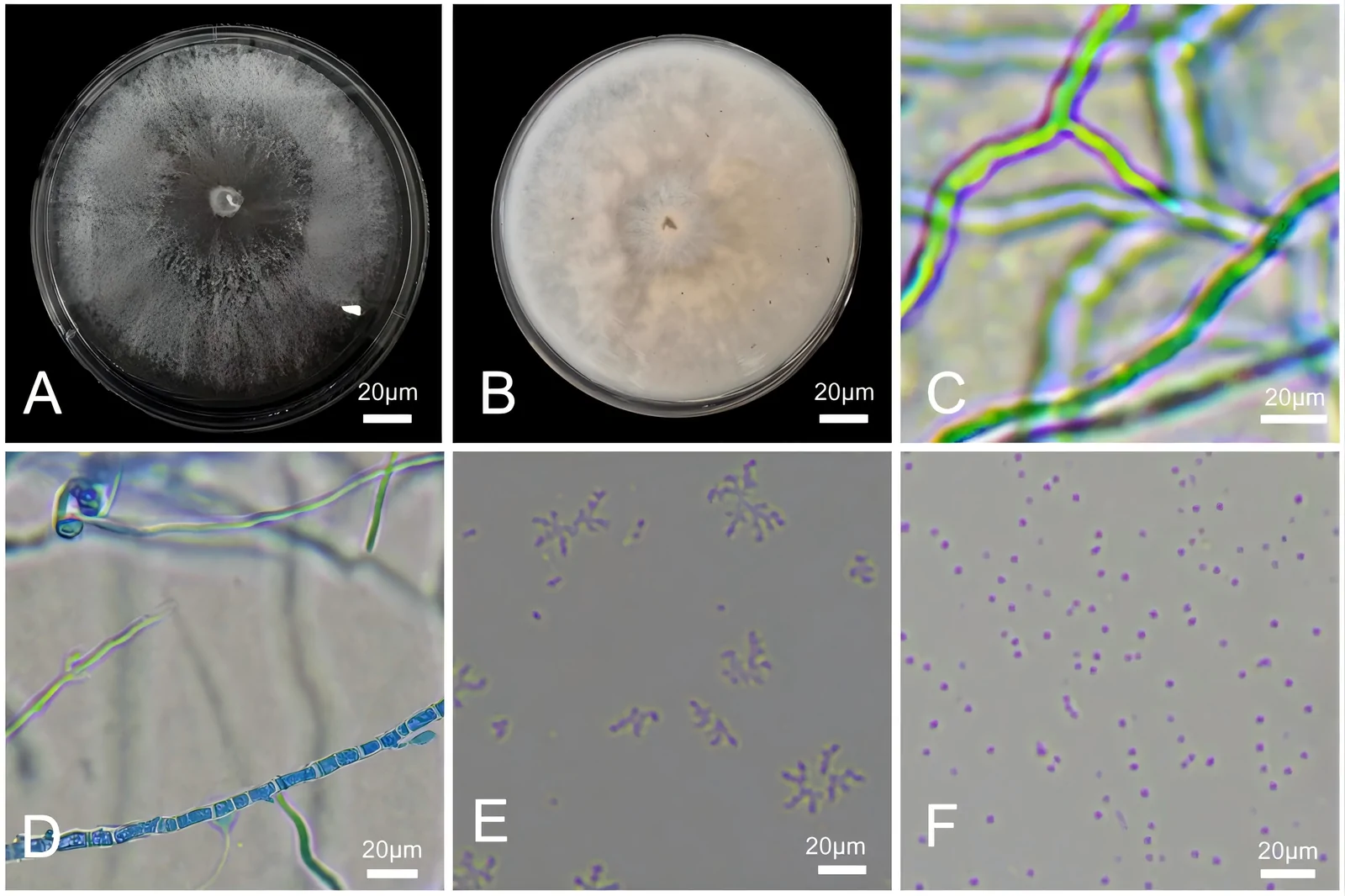
Colony morphology and microscopic characteristics of isolate B4. **(A, B)** Obverse and reverse colony morphology of isolate B4 incubated on PDA for 7 ad under light conditions. **(C)** Hyphal morphology. **(D)** Septate hyphae. **(E)** Conidiophores. **(F)** Conidia.

After 7 ad of incubation on PDA, colonies of isolate B4 were grayish white and flocculent, with abundant aerial mycelia and distinct radial growth patterns. The colony margins were regular, and small, raised sporulating structures were observed in the central region. The reverse side of the colonies was pale white to light beige, with slight diffusion of yellow pigment. Microscopic examination showed that the hyphae were hyaline, branched, and septate. The conidiophores were also hyaline and septate, with a loosely branched structure. The main conidiophore stalks were 12.8–33.2 μm long and 2.9–4.6 μm wide, with mean dimensions of 23.0 × 3.7 μm. Each main stalk produced 2–8 secondary branches, with an average of 5.0 branches. The secondary branches measured 3.8–8.8 μm in length and 2.6–4.8 μm in width. Hyphal fragments were 59.7–207.5 μm long and 4.8–7.8 μm wide. The conidia were elliptical and measured 4.4–7.6 × 2.9–5.4 μm, with a mean size of 6.0 × 4.1 μm. These morphological characteristics were consistent with those commonly observed in the genus *Diaporthe* (**Figure 2**).

### 3.3 Molecular identification of isolate B4

**Figure 3:**
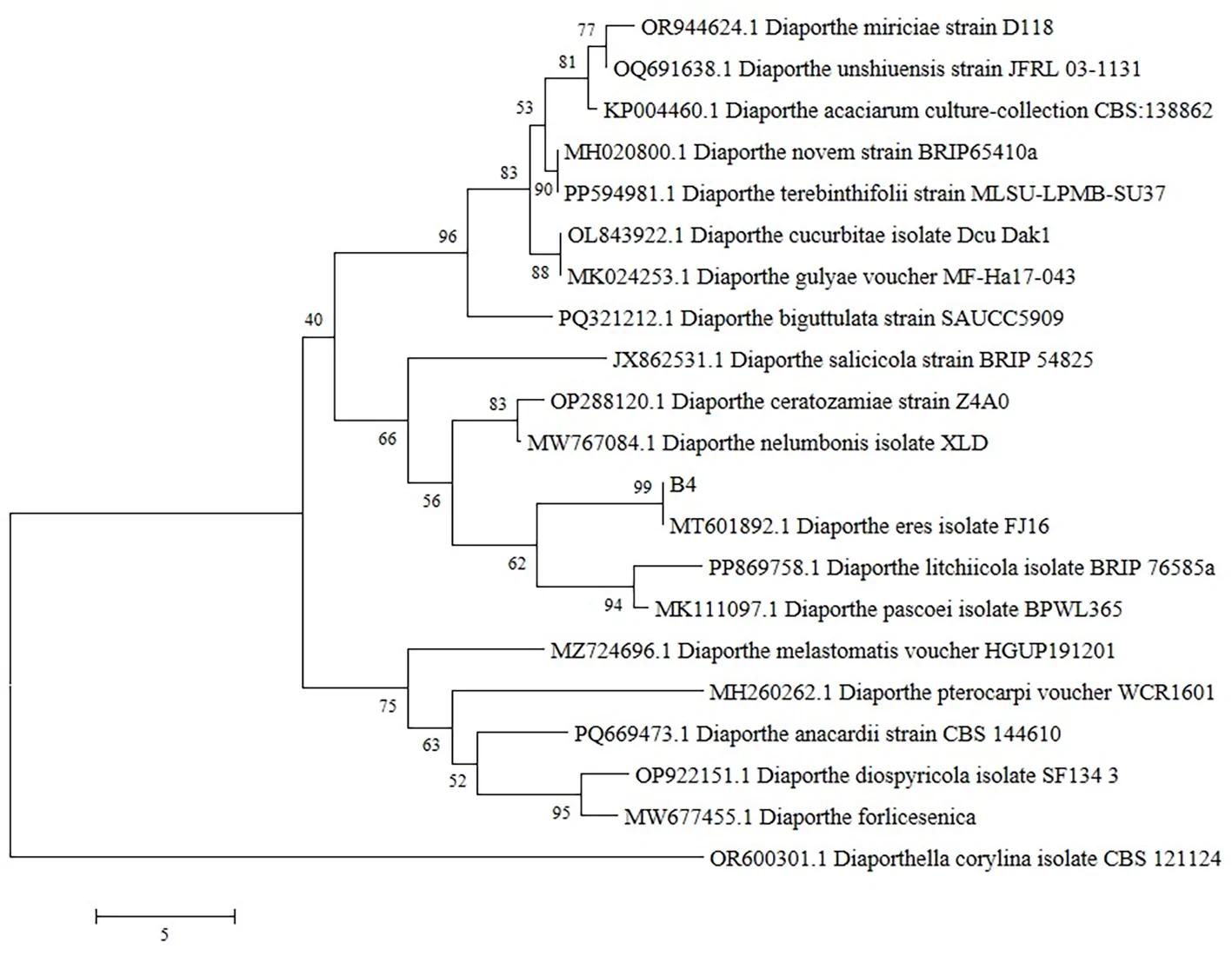
Neighbor-joining phylogenetic tree based on ITS sequences showing the phylogenetic placement of Diaporthe sp. isolate B4 among reference Diaporthe taxa. Bootstrap values from 1,000 replicates are shown at the nodes. The scale bar represents five nucleotide substitutions. GenBank accession numbers are provided after the reference taxon names.

ITS sequence analysis and phylogenetic reconstruction were performed to clarify the taxonomic placement of isolate B4. Based on its morphological characteristics and ITS sequence data, isolate B4 was assigned to the genus *Diaporthe*. In the neighbor-joining phylogenetic tree, isolate B4 was positioned near the reference isolate *D. eres* FJ16. However, because identification was based primarily on the ITS region, which does not provide sufficient resolution for reliable species-level identification within the genus *Diaporthe*, no species-level assignment was made. The fungus was therefore conservatively designated as *Diaporthe* sp. isolate B4. Together with the pathogenicity results, the isolate was considered to be associated with preharvest soft rot of ‘Ancui’ hardy kiwi (**Figure 3**).

### 3.4 Biological characteristics of the *Diaporthe* isolate

#### 3.4.1 Effects of culture media on mycelial growth

**Figure 4:**
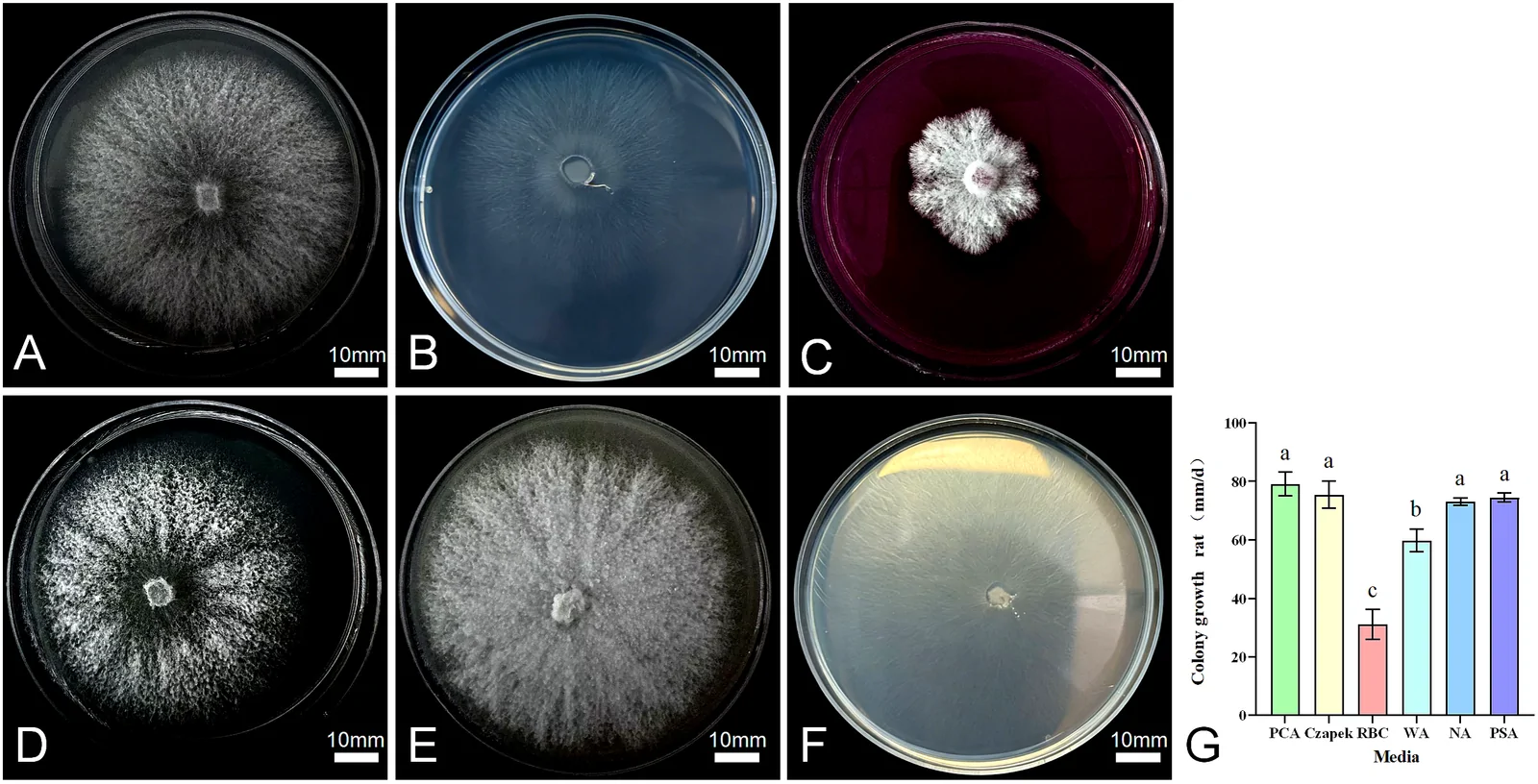
Effects of different culture media on the mycelial growth of *Diaporthe* sp. isolate B4, expressed as colony diameter after 7 ad of incubation. **(A)** Potato carrot agar (PCA). **(B)** Czapek’s agar. **(C)** Rose Bengal chloramphenicol agar (RBC). **(D)** Water agar (WA). **(E)** Nutrient agar (NA). **(F)** Potato sucrose agar (PSA). **(G)** Colony diameter statistics. Error bars represent ± SD (n = 3). Different lowercase letters indicate significant differences among treatments according to Duncan’s multiple range test at *P* < 0.05.

Mycelial growth varied among the six tested culture media. PCA, Czapek’s agar, NA, and PSA supported relatively vigorous colony growth, whereas growth on WA was moderate and RBC supported the least growth. Among the tested media, PCA was the most favorable, with the colony diameter reaching 79.10 mm after 7 ad of incubation (**Figure 4**).

#### 3.4.2 Effects of nitrogen sources on mycelial growth

**Figure 5:**
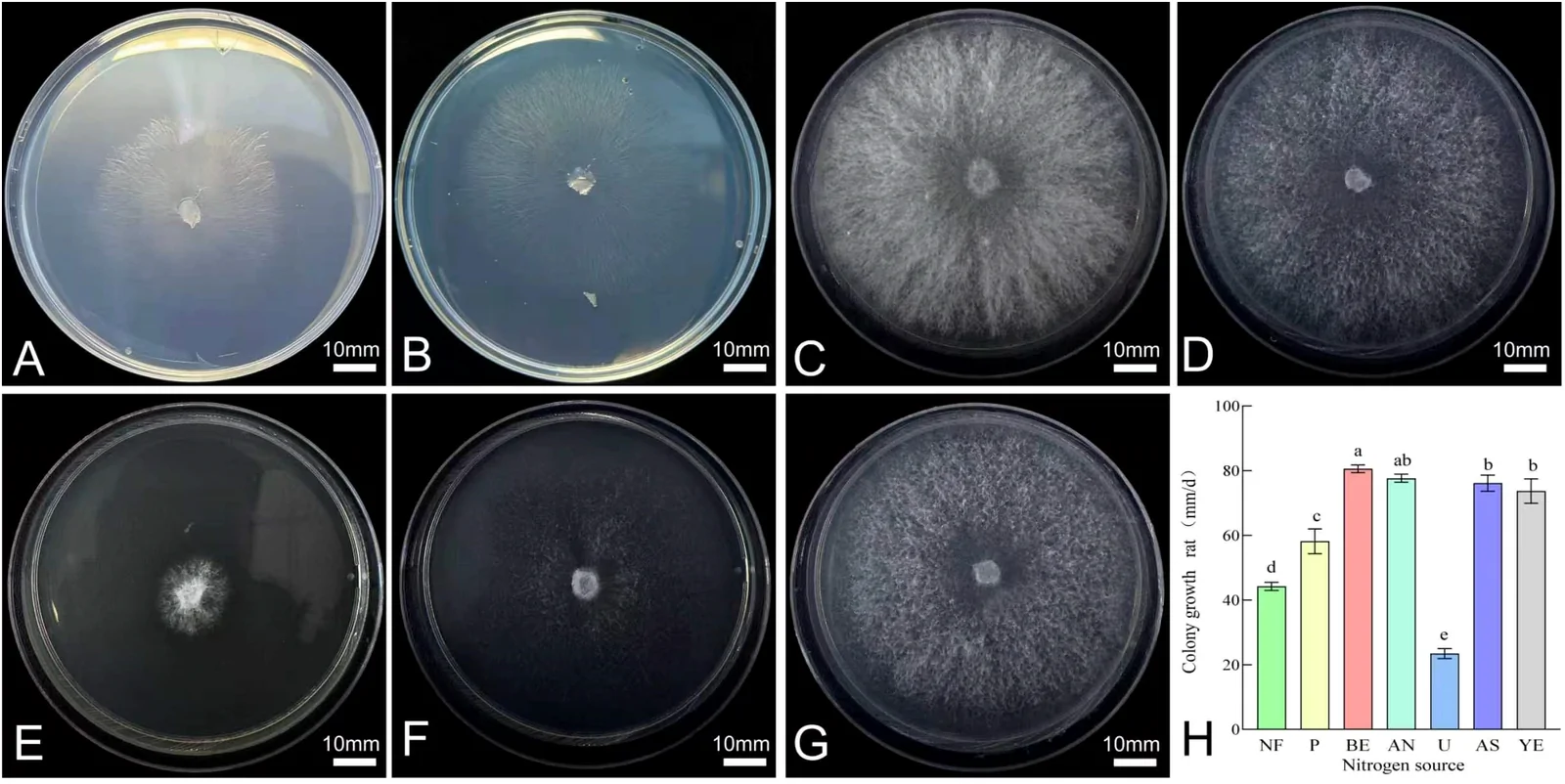
Effects of different nitrogen sources on the mycelial growth of *Diaporthe* sp. isolate B4, expressed as colony diameter after 7 ad of incubation. **(A)** Beef extract. **(B)** Peptone. **(C)** Yeast extract. **(D)** Ammonium sulfate. **(E)** Ammonium nitrate. **(F)** Urea. **(G)** Nitrogen-free control. **(H)** Colony diameter statistics. Error bars represent ± SD (n = 3). Different lowercase letters indicate significant differences among treatments according to Duncan’s multiple range test at *P* < 0.05.

Among the tested nitrogen sources, beef extract supported the greatest mycelial growth. After 7 ad of incubation, the colony diameter in the beef extract treatment reached 80.59 mm, with an average daily growth rate of 10.80 mm d^−1^. Growth on beef extract was comparable to that on ammonium nitrate and yeast extract and was greater than that on the other nitrogen sources. Urea markedly restricted colony development, whereas only limited growth was observed in the nitrogen-free control (**Figure 5**).

#### 3.4.3 Effects of carbon sources on mycelial growth

**Figure 6:**
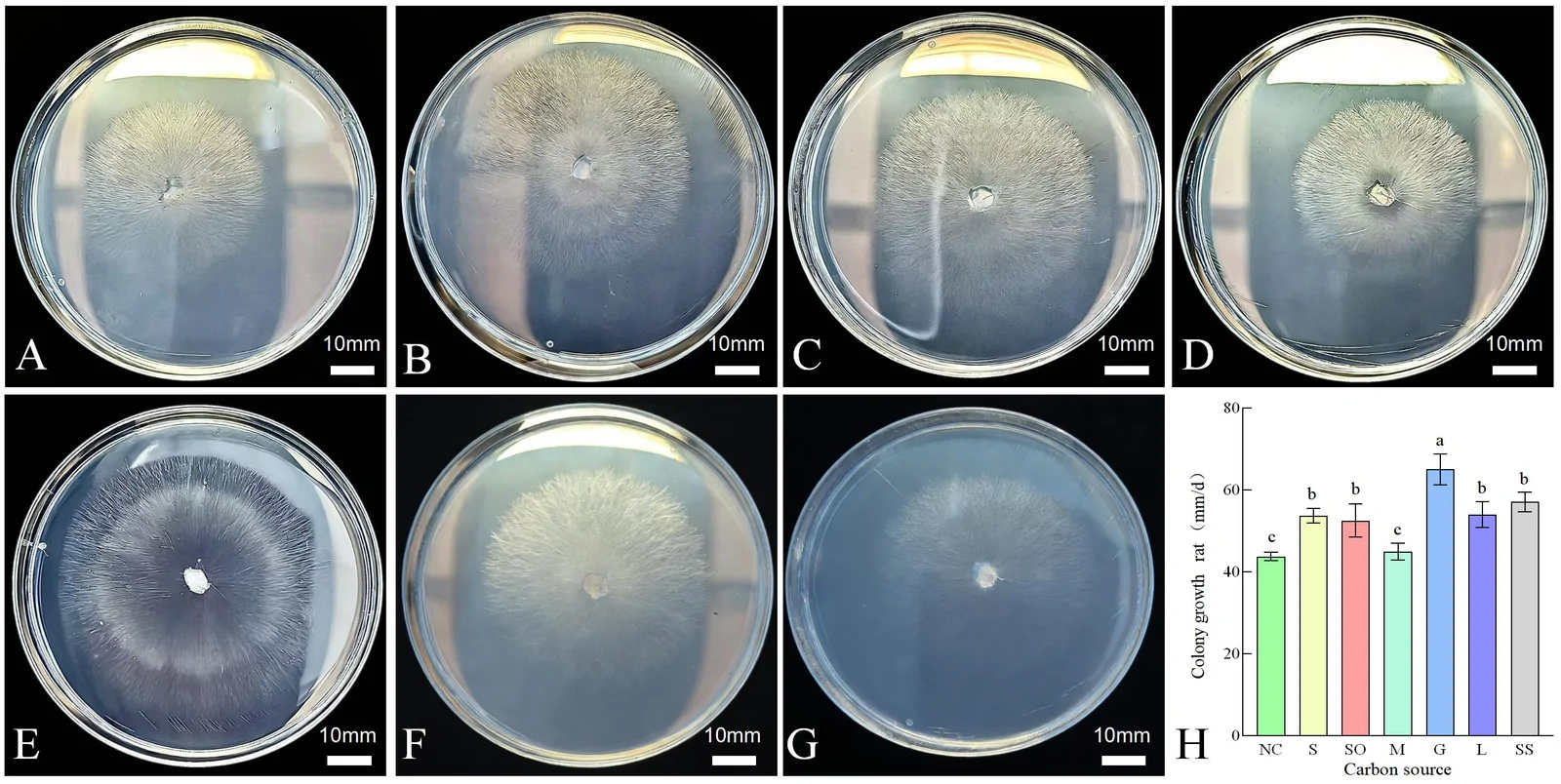
Effects of different carbon sources on the mycelial growth of *Diaporthe* sp. isolate B4, expressed as colony diameter after 7 ad of incubation. **(A)** Carbon-free control (NC). **(B)** Sucrose (S). **(C)** Sorbitol (SO). **(D)** Mannitol (M). **(E)** Glucose (G). **(F)** Lactose (L). **(G)** Soluble starch (SS). **(H)** Colony diameter statistics. Error bars represent ± SD (n = 3). Different lowercase letters indicate significant differences among treatments according to Duncan’s multiple range test at *P* < 0.05.

Glucose supported the greatest mycelial growth among the tested carbon sources. After 7 ad of incubation, the colony diameter in the glucose treatment reached 65.05 mm, with an average daily growth rate of 8.58 mm d^−1^. Sucrose, sorbitol, lactose, and soluble starch supported moderate colony growth, whereas mannitol and the carbon-free control restricted mycelial development. These results identified glucose as the most favorable carbon source under the tested conditions (**Figure 6**).

#### 3.4.4 Effects of light conditions on mycelial growth

**Figure 7:**
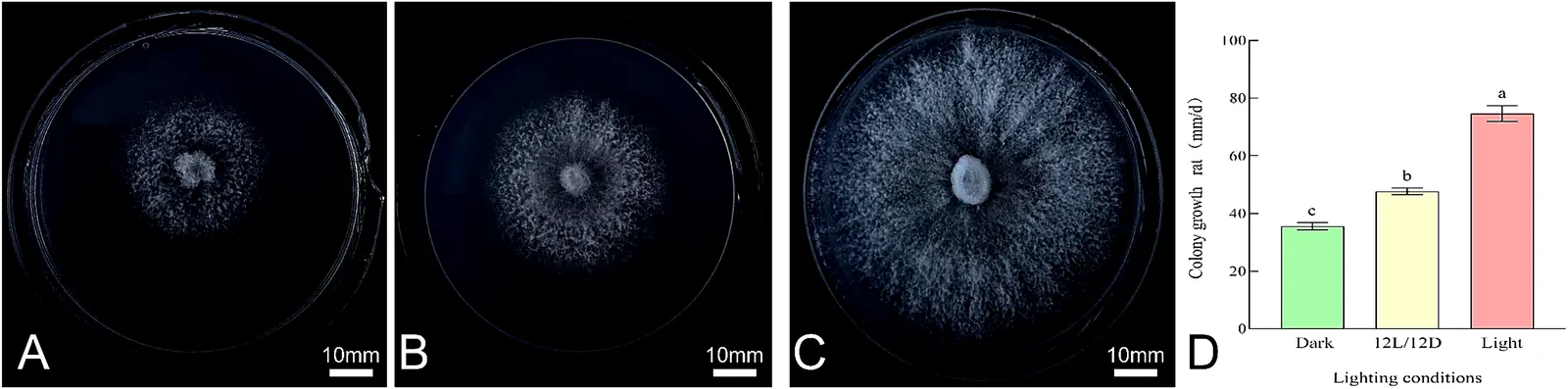
Effects of different light conditions on colony morphology and mycelial growth of *Diaporthe* sp. isolate B4, expressed as colony diameter after 7 ad of incubation. **(A)** Complete darkness. **(B)** 12 ah light/12 ah dark alternation. **(C)** Continuous light. **(D)** Colony diameter statistics. Error bars represent ± SD (n = 3). Different lowercase letters indicate significant differences among treatments according to Duncan’s multiple range test at *P* < 0.05.

Continuous light produced the greatest colony expansion, whereas continuous darkness restricted mycelial growth. Growth under the 12 ah light/12 ah dark regime was intermediate between these two treatments. These results indicated that light exposure favored the vegetative growth of isolate B4 under the tested culture conditions (**Figure 7**).

#### 3.4.5 Effects of temperature on mycelial growth

**Figure 8:**
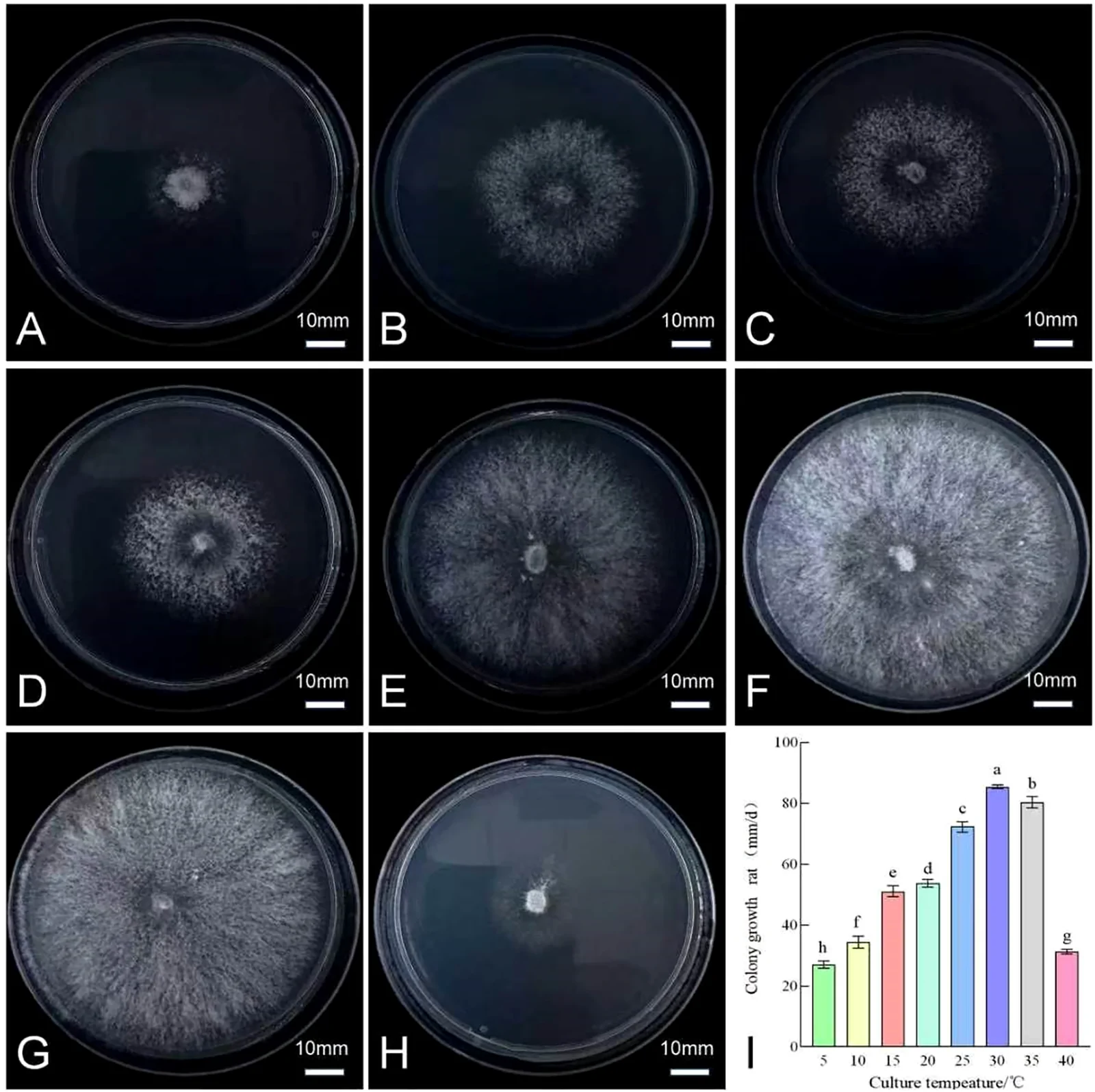
Effects of different temperatures on colony morphology and mycelial growth of *Diaporthe* sp. isolate B4, expressed as colony diameter after 7 ad of incubation. **(A)** 5 °C. **(B)** 10 °C. **(C)** 15 °C. **(D)** 20 °C. **(E)** 25 °C. **(F)** 30 °C. **(G)** 35 °C. **(H)** 40 °C. Error bars represent ± SD (n = 3). Different lowercase letters indicate significant differences among treatments according to Duncan’s multiple range test at *P* < 0.05.

Mycelial growth varied markedly across the tested temperatures. Growth was strongly restricted at 5 °C and gradually increased as the temperature rose. The isolate grew vigorously at 25–35 °C, with the maximum colony diameter observed at 30 °C. After 7 ad of incubation at 30 °C, the colony diameter reached 85.00 mm and the average daily growth rate was 11.43 mm d^−1^. Therefore, 30 °C was considered the optimum temperature under the tested conditions (**Figure 8**).

#### 3.4.6 Effects of pH on mycelial growth

**Figure 9:**
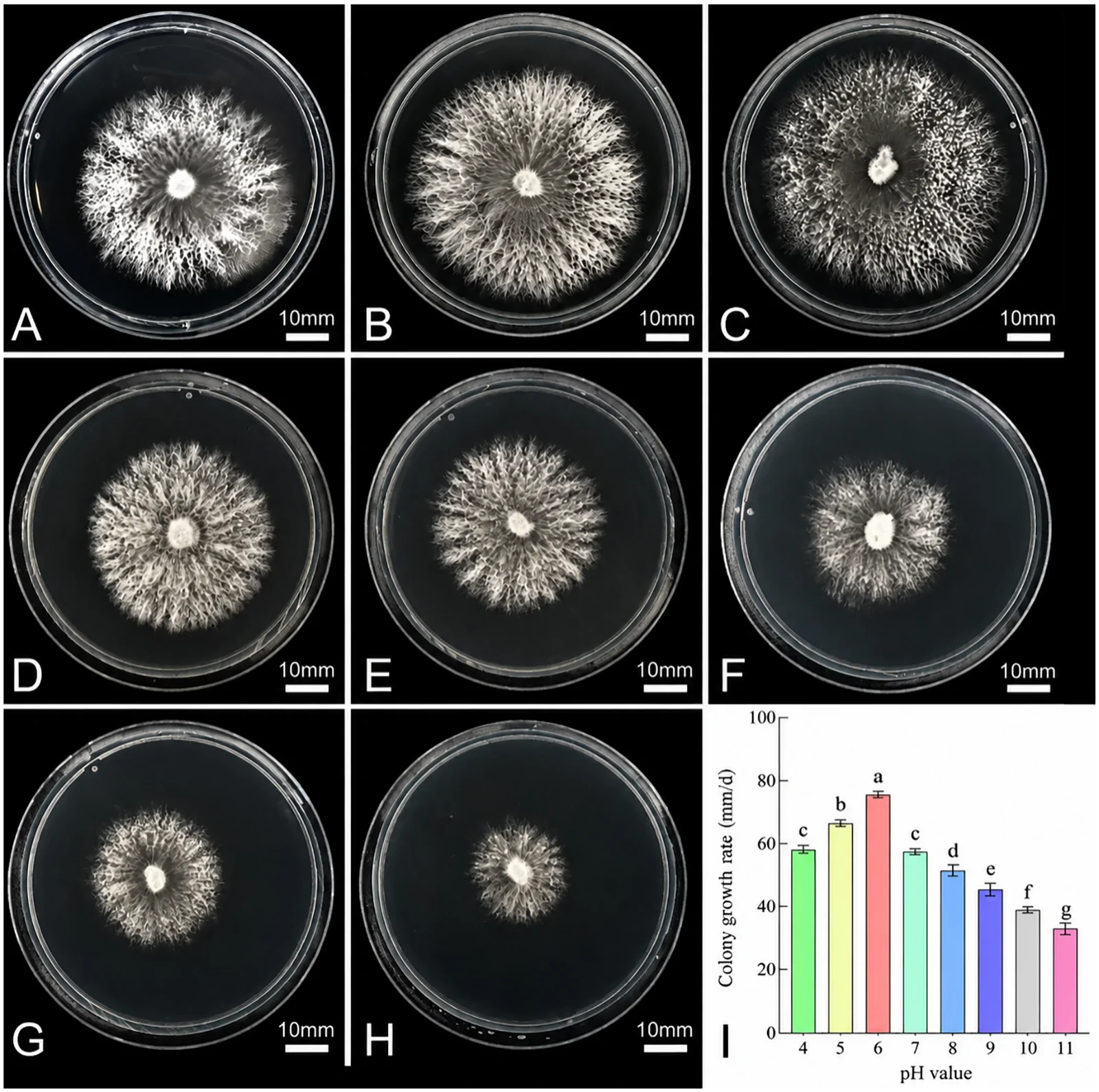
Effects of different pH values on colony morphology and mycelial growth of *Diaporthe* sp. isolate B4. **(A)** pH 4. **(B)** pH 5. **(C)** pH 6. **(D)** pH 7. **(E)** pH 8. **(F)** pH 9. **(G)** pH 10. **(H)** pH 11. **(I)** Colony diameter statistics. Error bars represent ± SD (n = 3). Different lowercase letters above the bars indicate significant differences among treatments according to Duncan’s multiple range test at *P* < 0.05.

The isolate grew throughout the tested pH range of 4–11, demonstrating relatively broad pH adaptability. The greatest mycelial growth was observed at pH 6, whereas strongly acidic and alkaline conditions restricted colony expansion. Thus, weakly acidic conditions were more favorable for the growth of isolate B4, with pH 6 representing the optimum value under the tested conditions (**Figure 9**).

### 3.5 *In vitro* antifungal activity and toxicity regression analysis of allicin against *Diaporthe* sp. isolate B4

**Figure 10:**
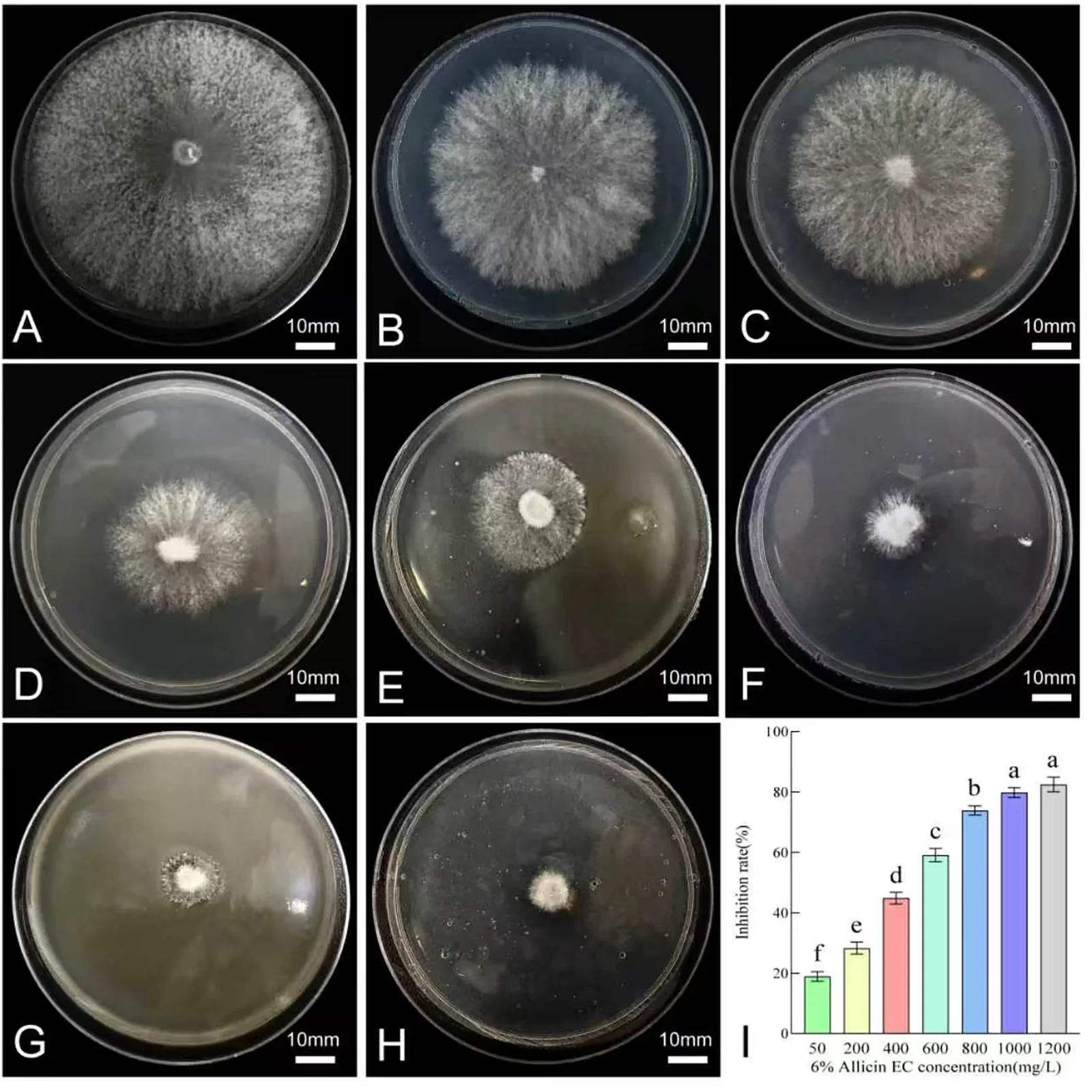
Effects of different concentrations of 6% allicin EC on the mycelial growth of *Diaporthe* sp. isolate B4 and inhibition rate after 7 ad of incubation. **(A)** Blank control (CK). **(B)** 50 mg/L. **(C)** 200 mg/L. **(D)** 400 mg/L. **(E)** 600 mg/L. **(F)** 800 mg/L. **(G)** 1,000 mg/L. **(H)** 1,200 mg/L. **(I)** Inhibition rate statistics. Error bars represent ± SD (n = 3). Different lowercase letters indicate significant differences among treatments according to Duncan’s multiple range test at *P* < 0.05.

The mycelial growth of *Diaporthe* sp. isolate B4 was progressively suppressed as the allicin concentration increased (**Figure 10**). The blank control exhibited vigorous colony growth, whereas the mean inhibition rate increased from 18.91% at 50 mg/L to 82.52% at 1,200 mg/L. The inhibition rates at 50, 200, and 400 mg/L were 18.91%, 28.32%, and 44.85%, respectively. At 600, 800, 1,000, and 1,200 mg/L, the inhibition rates increased to 59.09%, 73.87%, 79.86%, and 82.52%, respectively.

**Figure 11:**
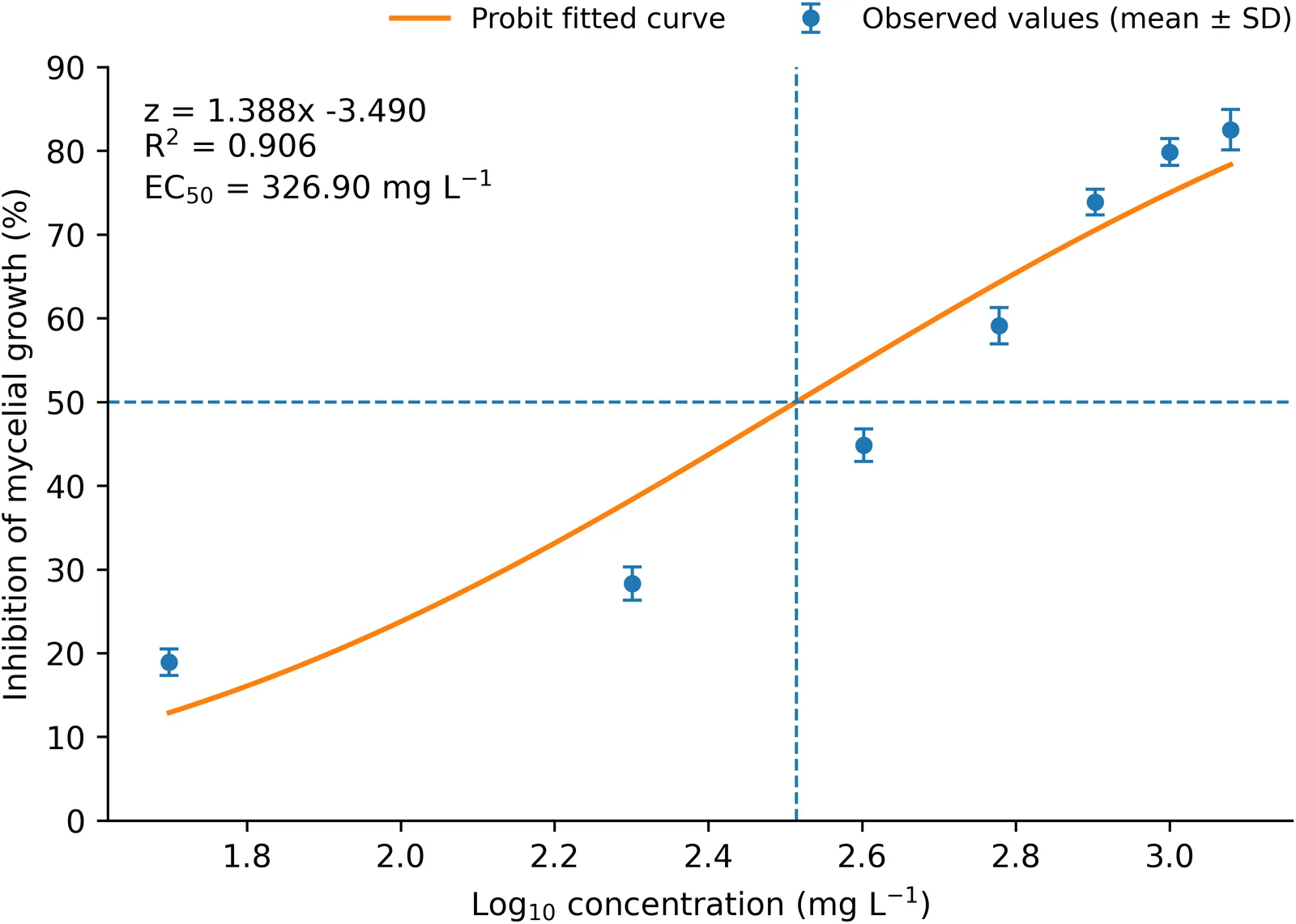
Concentration–response relationship of 6% allicin EC against *Diaporthe* sp. isolate B4. Points represent the observed mean inhibition rates ± SD (n = 3), and the fitted line represents the probit regression model. Dashed lines indicate the estimated EC_50_ value of 326.90 mg/L.

Probit regression revealed a linear relationship between the log_10_-transformed allicin concentration and the inhibition response (**Figure 11**; **Table 1**). The fitted regression equation was z = 1.388x − 3.490, with an R^2^ value of 0.906. The estimated EC_50_ and EC_90_ values were 326.90 and 2,739.29 mg/L, respectively. The corresponding 95% confidence intervals were 242.29–441.07 mg/L for EC_50_ and 1,498.78–5,006.55 mg/L for EC_90_.

**Table 1:** Probit regression analysis of 6% allicin EC against *Diaporthe* sp. isolate B4.

Parameter	Value
Regression equation	z = 1.388x − 3.490
R^2^	0.906
EC_50_ (mg/L)	326.90
95% CI of EC_50_	242.29–441.07
EC_90_ (mg/L)	2,739.29
95% CI of EC_90_	1,498.78–5,006.55

x represents log10-transformed concentration, and z represents the standard-normal probit value.

### 3.6 Field control efficacy of allicin against preharvest soft rot

Before the first application, the disease index ranged from 2.71 to 2.93 among treatments, with no significant differences detected, indicating relatively uniform initial disease severity in the field (**Table 2**). After the first application, control efficacy generally increased with increasing allicin concentration. The 50 and 200 mg/L treatments showed relatively limited control efficacy, reaching 18.08% and 31.92%, respectively. Greater control efficacy was observed at concentrations of 400–1,200 mg/L, and the highest efficacy after the first application was obtained at 1,000 mg/L, reaching 78.07%. Control efficacy was numerically higher after the second application at all allicin concentrations. The highest efficacy was observed at 1,000 mg/L, reaching 81.92%, followed by 800 mg/L at 80.09%. No significant difference was detected between the 800 and 1,000 mg/L treatments. The 1,200 mg/L treatment showed a control efficacy of 78.13%, which was significantly lower than that of the 1,000 mg/L treatment but was not significantly different from that of the 800 mg/L treatment, indicating that disease suppression remained high despite the modest decline at the highest concentration tested. In contrast, the disease index of the sterile-water control increased to 19.26 after the second application. Overall, 800–1,000 mg/L represented the most effective concentration range for controlling preharvest soft rot under the conditions of this field trial.

**Table 2:** Field control efficacy of 6% allicin EC against preharvest soft rot of ‘Ancui’ hardy kiwi.

Agent	Conc. (mg·L^−1^)	Pre-treatment DI	7 d after 1st spray		7 d after 2nd spray	
			DI	CE (%)	DI	CE (%)
6% allicin EC	50	2.74 ± 0.1a	14.81 ± 0.7b	18.08 ± 0.2g	12.71 ± 0.8b	28.85 ± 2.4f
	200	2.93 ± 0.2a	12.34 ± 0.8c	31.92 ± 0.2f	11.11 ± 0.7c	39.14 ± 0.9e
	400	2.71 ± 0.4a	10.86 ± 1.3d	40.02 ± 0.2e	10.00 ± 0.7d	47.16 ± 0.9d
	600	2.78 ± 0.2a	7.04 ± 0.5e	68.06 ± 0.2d	6.30 ± 0.4e	72.12 ± 1.0c
	800	2.84 ± 0.3a	5.27 ± 0.2f	75.12 ± 0.2b	4.67 ± 0.2f	80.09 ± 0.8ab
	1000	2.78 ± 0.2a	4.80 ± 0.1f	78.07 ± 0.2a	4.20 ± 0.1f	81.92 ± 0.74a
	1200	2.78 ± 0.1a	5.30 ± 0.1f	74.09 ± 0.2c	4.80 ± 0.1f	78.13 ± 0.9b
CK		2.78 ± 0.1a	16.67 ± 0.4a		19.26 ± 0.4a	

Values are presented as the mean ± SD of three biological replicates. Different lowercase letters within the same column indicate significant differences among treatments according to Duncan’s multiple range test at *P* < 0.05. CK represents the sterile-water control. DI, disease index; CE, control efficacy.

### 3.7 Effects of allicin on oxidative stress indicators in hardy kiwi fruit

**Figure 12:**
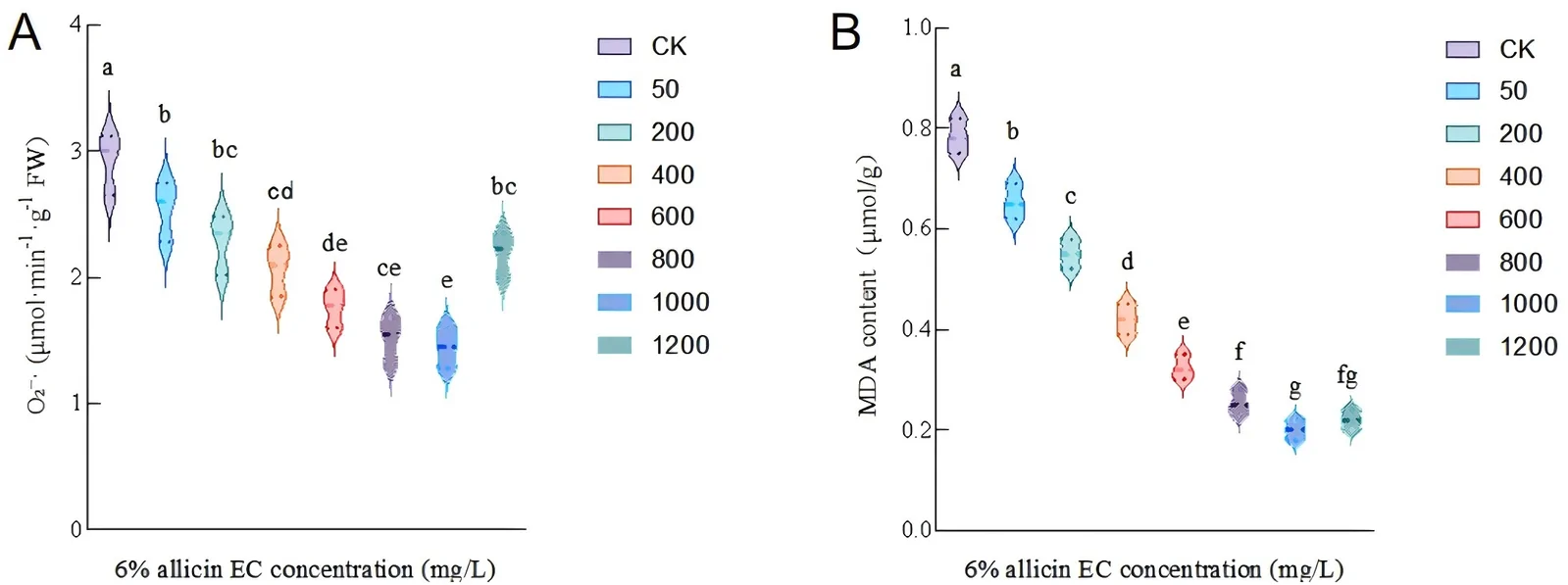
Effects of 6% allicin EC on oxidative stress indicators in ‘Ancui’ hardy kiwi fruit during soft rot development. **(A)** Superoxide anion (O_2_•^−^) production rate. **(B)** Malondialdehyde (MDA) content. Error bars represent ± SD (n = 3). Different lowercase letters indicate significant differences among treatments according to Duncan’s multiple range test at *P* < 0.05.

Allicin treatments generally reduced oxidative stress indicators in ‘Ancui’ hardy kiwi fruit (**Figure 12**). The O_2_•^−^ production rate was relatively high in the sterile-water control, whereas allicin treatments generally reduced O_2_•^−^ accumulation. With increasing allicin concentration, the O_2_•^−^ production rate initially decreased and then increased slightly at higher concentrations. The lowest O_2_•^−^ production rate was observed at 1,000 mg/L, indicating that moderate-to-high allicin concentrations were associated with reduced O_2_•^−^ accumulation in fruit tissues.

MDA content showed a response pattern similar to that of O_2_•^−^. Compared with the sterile-water control, allicin treatments significantly reduced MDA accumulation, suggesting reduced membrane lipid peroxidation during disease development. MDA content decreased with increasing allicin concentration and reached its lowest level at 1,000 mg/L. At 1,200 mg/L, MDA content increased slightly compared with that at 1,000 mg/L but remained lower than that in the control. These results indicated that allicin treatment reduced oxidative damage in hardy kiwi fruit, with the greatest reduction observed at approximately 1,000 mg/L.

### 3.8 Effects of allicin on defense-related enzyme activities in hardy kiwi fruit

**Figure 13:**
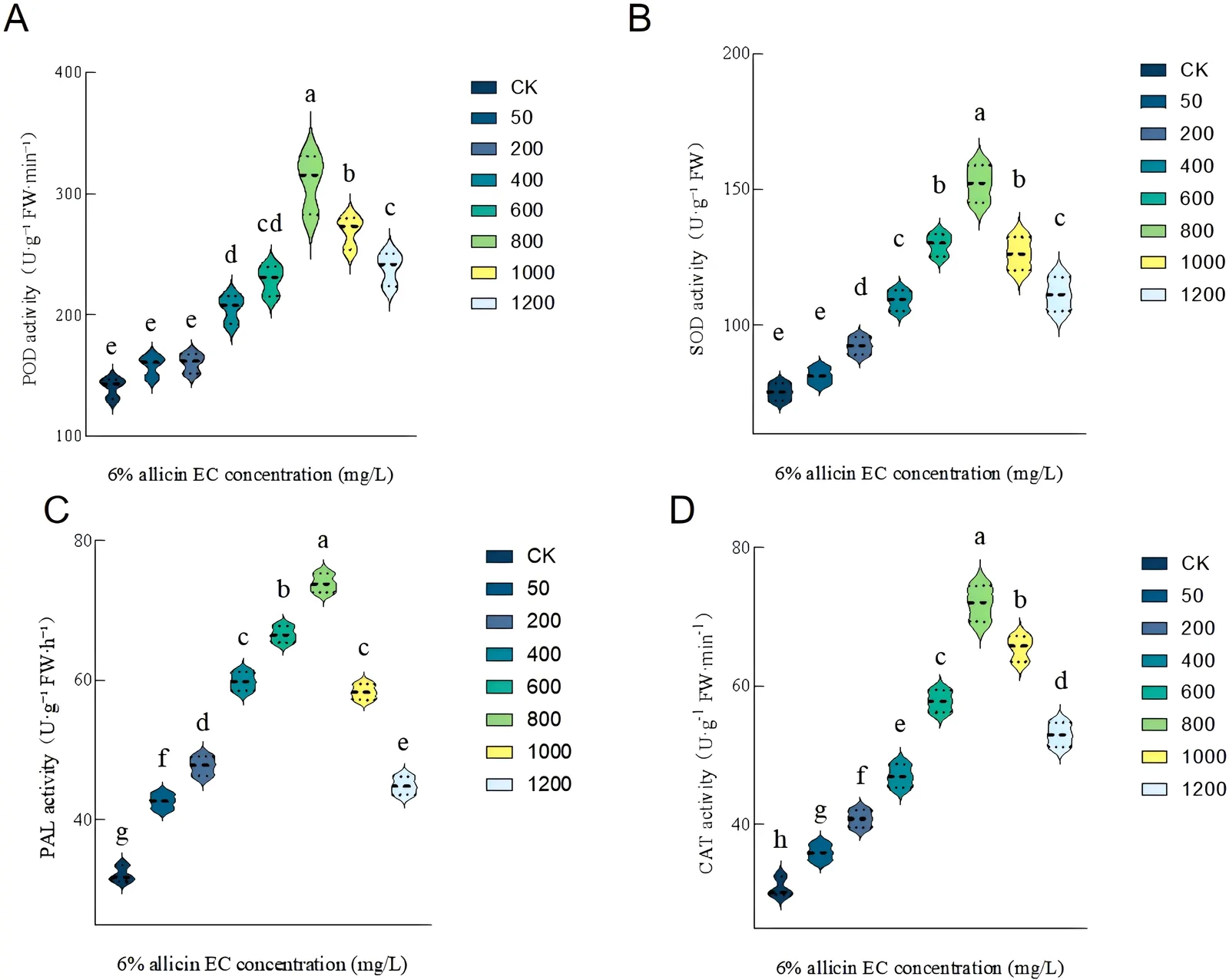
Effects of 6% allicin EC on defense-related enzyme activities in ‘Ancui’ hardy kiwi fruit during soft rot development. **(A)** Peroxidase (POD) activity. **(B)** Superoxide dismutase (SOD) activity. **(C)** Phenylalanine ammonia-lyase (PAL) activity. **(D)** Catalase (CAT) activity. Error bars represent ± SD (n = 3). Different lowercase letters indicate significant differences among treatments according to Duncan’s multiple range test at *P* < 0.05.

The activities of POD, SOD, PAL, and CAT generally increased with increasing allicin concentration and then declined at higher concentrations (**Figure 13**). Compared with the sterile-water control, all allicin treatments significantly increased POD activity. The highest POD activity was observed at 800 mg/L, reaching approximately 221.4% of the control level. POD activity gradually declined at 1,000 and 1,200 mg/L but remained higher than that in the low-concentration treatments and the sterile-water control. SOD activity exhibited a similar response pattern. All allicin treatments significantly enhanced SOD activity relative to the sterile-water control, with the maximum activity recorded at 800 mg/L, reaching approximately 214.3% of the control level. The increase in SOD activity was less pronounced at lower concentrations, indicating that low allicin concentrations induced a comparatively weaker antioxidant response.

PAL activity also initially increased and subsequently decreased as the allicin concentration increased. The highest PAL activity was observed at 800 mg/L, reaching approximately 231.0% of the control level. PAL activity declined at higher concentrations, and the response at 1,200 mg/L was weaker than those at 800 and 1,000 mg/L. CAT activity followed the same general pattern, with intermediate allicin concentrations producing a greater increase than the lower concentrations or the highest concentration tested. Overall, the enhancement of antioxidant and defense-related enzyme activities was most pronounced at 800–1,000 mg/L, indicating that this concentration range was associated with stronger defense-related physiological responses in hardy kiwi fruit.

### 3.9 Correlation analysis between field control efficacy and physiological responses

**Figure 14:**
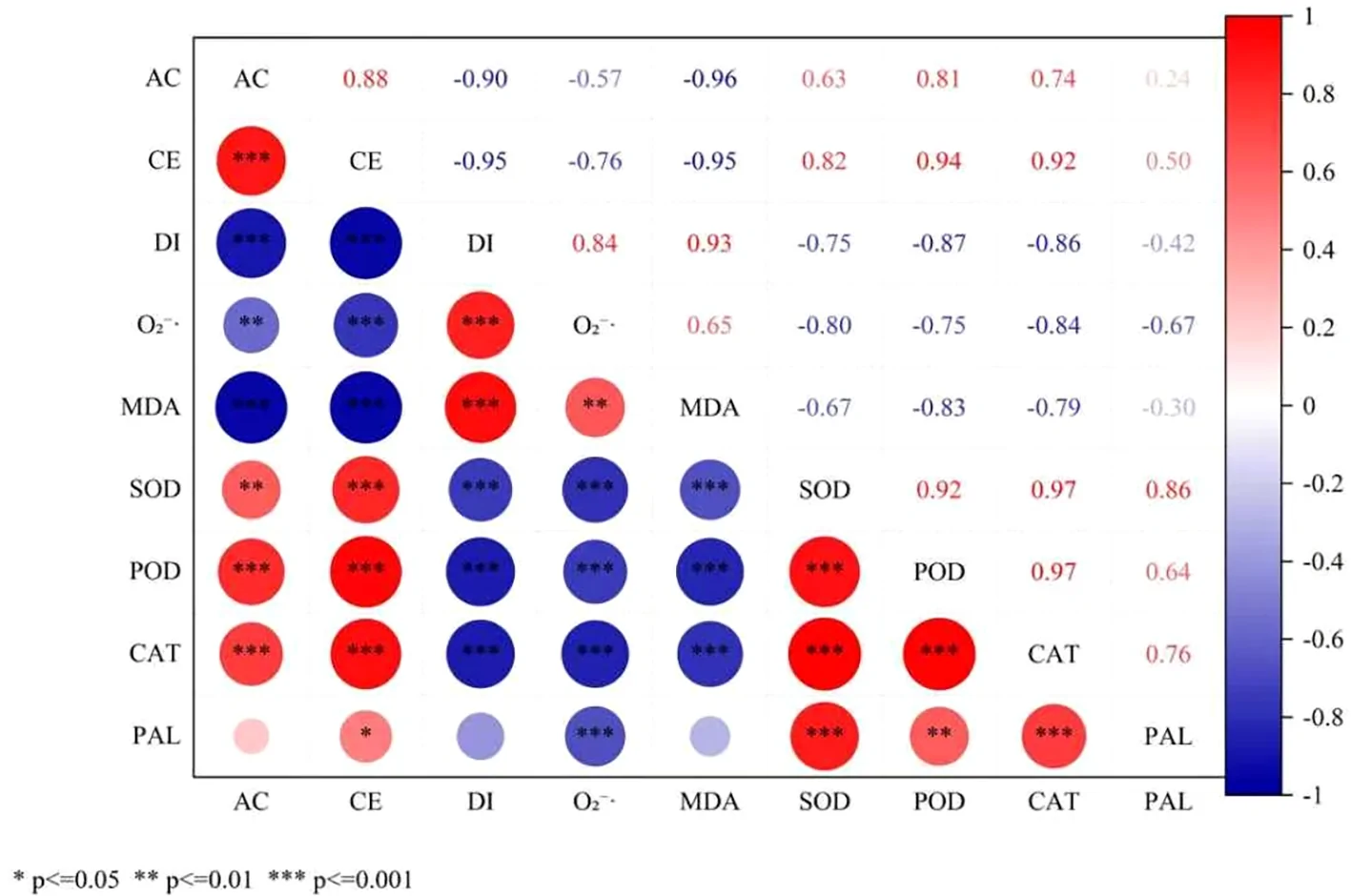
Spearman correlation heatmap among allicin concentration, field control efficacy, disease index, oxidative stress indicators, and defense-related enzyme activities in *A. arguta* fruits treated with 6% allicin EC. Red and blue indicate positive and negative correlations, respectively. Circle size represents correlation strength. *, **, and *** indicate significance at *P* < 0.05, *P* < 0.01, and *P* < 0.001, respectively. AC, allicin concentration; CE, control efficacy; DI, disease index; O_2_•^−^, superoxide anion; MDA, malondialdehyde; SOD, superoxide dismutase; POD, peroxidase; CAT, catalase; PAL, phenylalanine ammonia-lyase.

Spearman correlation analysis revealed clear relationships among field control efficacy, disease index, oxidative stress indicators, and defense-related enzyme activities (**Figure 14**). Field control efficacy was negatively correlated with disease index, O_2_•^−^ production rate, and MDA content but positively correlated with SOD, POD, CAT, and PAL activities. In contrast, disease index was positively correlated with O_2_•^−^ production rate and MDA content but negatively correlated with defense-related enzyme activities. These results indicated that higher field control efficacy was associated with lower disease severity and oxidative damage and with greater defense-related enzyme activities. The correlation analysis further supported the association between allicin-mediated disease suppression and defense-related physiological responses in hardy kiwi fruit.

## 4 Discussion

Preharvest soft rot is an important disease affecting the production and marketability of hardy kiwi fruit. ‘Ancui’ is a newly bred hardy kiwi cultivar with promising cultivation potential in the cold northern regions of China; however, information on its disease-associated pathogens and effective management strategies remains limited. In this study, a fungal isolate was obtained from symptomatic ‘Ancui’ hardy kiwi fruit collected in Hunjiang District. Morphological characteristics, ITS sequence analysis, and phylogenetic placement supported the assignment of isolate B4 to the genus *Diaporthe*. Although isolate B4 was positioned near *D. eres* isolate FJ16 in the ITS-based phylogenetic tree, this ITS-based relationship was not considered sufficient for definitive species-level identification. Therefore, the fungus was conservatively designated as *Diaporthe* sp. isolate B4 rather than assigned to a named species. Reliable species-level identification within *Diaporthe* would require sequencing of additional gene loci and multilocus phylogenetic analysis. Artificial inoculation reproduced typical soft rot symptoms, and a morphologically consistent fungus was re-isolated from the symptomatic tissues, fulfilling Koch’s postulates. These findings confirmed that *Diaporthe* sp. isolate B4 was pathogenic to ‘Ancui’ hardy kiwi and associated with preharvest soft rot. Previous studies have reported *Diaporthe* species in association with fruit rot and other diseases of cultivated kiwifruit and several fruit trees (Shi et al., 2020; Lyu, 2024; Qiao et al., 2024). However, information on the association of *Diaporthe* species with preharvest soft rot of the newly bred hardy kiwi cultivar ‘Ancui’ remains limited. The present study therefore expands the current understanding of fungal pathogens affecting this cultivar and provides a basis for developing targeted disease-management strategies.

The biological characteristics of *Diaporthe* sp. isolate B4 demonstrated relatively broad adaptability to different nutritional and environmental conditions. The isolate grew well on several culture media, particularly PCA, while beef extract and glucose were the most favorable nitrogen and carbon sources, respectively. Mycelial growth occurred throughout the tested pH range of 4–11 and was greatest at pH 6, indicating a preference for weakly acidic conditions. The isolate also grew vigorously at 25–35 °C, with the greatest growth observed at approximately 30 °C. However, these values represent the optimal conditions for mycelial growth *in vitro* rather than strict environmental thresholds for disease occurrence in the orchard. During the field trial, the mean temperature ranged from 22 to 28 °C and the relative humidity ranged from 65% to 85%; this temperature range partially overlapped with the range supporting vigorous mycelial growth.

The concentrated occurrence of soft rot during late fruit development is therefore likely to reflect the combined effects of pathogen adaptability, fruit maturity, and the orchard microenvironment rather than temperature or pH alone. Previous studies have shown that the physicochemical characteristics of hardy kiwifruit, including fruit firmness, acidity, and pH, change during fruit ripening (Fisk et al., 2008). Such maturity-associated changes, particularly progressive tissue softening, may weaken the structural barriers that restrict fungal invasion. In addition, *Diaporthe* isolates associated with kiwifruit rot can secrete pectinases and cellulases that contribute to cell-wall degradation during infection (Ling et al., 2024). Moisture retained around the fruit stalk, natural openings, or minor injuries may further favor infection and symptom development under humid orchard conditions. Thus, the broad temperature and pH adaptability of isolate B4 may facilitate its survival in the orchard, whereas maturity-associated tissue susceptibility and favorable moisture conditions may jointly contribute to the increased occurrence of soft rot during late fruit development.

The *in vitro* assay showed that 6% allicin EC inhibited the mycelial growth of *Diaporthe* sp. isolate B4 in a concentration-dependent manner. Probit regression further quantified this inhibitory effect, yielding estimated EC_50_ and EC_90_ values of 326.90 and 2,739.29 mg/L, respectively. The inhibition rate exceeded 50% at 600 mg/L and continued to increase at higher concentrations, indicating that allicin exerted direct antifungal activity against the isolate under controlled laboratory conditions. Allicin is a sulfur-containing antimicrobial compound derived from garlic and has been reported to inhibit a range of fungal and bacterial pathogens (Curtis et al., 2004; Portz et al., 2008; Kim et al., 2012; Hayat et al., 2016; Leontiev et al., 2018; Zhang et al., 2020; Tan and Tao, 2024). Previous studies on kiwifruit rot and biological control have also shown that plant extracts, garlic-derived compounds, antagonistic microorganisms, and microbial metabolites can suppress fungal pathogens or reduce fruit decay (Qiao, 2025; Qiao et al., 2024; Fan et al., 2023; Li et al., 2021). The present findings extend these observations by demonstrating the inhibitory activity of allicin against a *Diaporthe* isolate associated with preharvest soft rot of the newly bred hardy kiwi cultivar ‘Ancui’ and support its potential as a botanical antifungal agent for disease management.

Under field conditions, allicin treatment effectively reduced the development of preharvest soft rot in ‘Ancui’ hardy kiwi, with the highest control efficacy observed at 800–1,000 mg/L. After the second application, control efficacy reached 80.09% at 800 mg/L and 81.92% at 1,000 mg/L, with no significant difference between these two treatments. These results indicate that allicin provided substantial disease suppression under the conditions of this orchard trial and support its potential for further field evaluation against preharvest soft rot. The apparent difference between the laboratory EC_50_ value of 326.90 mg/L and the optimal field application range of 800–1,000 mg/L should be interpreted in light of the different biological endpoints and exposure conditions used in the two assays. The EC_50_ represents the formulation concentration required to inhibit mycelial growth by 50% under continuous and relatively uniform contact on allicin-amended PDA, whereas field control efficacy reflects the suppression of disease development on intact fruits under natural infection conditions. Indeed, 800 and 1,000 mg/L produced *in vitro* inhibition rates of 73.87% and 79.86%, respectively, which were more comparable to the field control efficacies of 80.09% and 81.92% after the second application than to the 50% inhibition represented by the EC_50_. Under orchard conditions, spray deposits may be distributed unevenly and reduced by runoff, rainfall, light exposure, temperature fluctuations, and degradation or loss of active compounds. Consequently, a formulation concentration higher than the laboratory EC_50_ may be required to maintain sufficient exposure on the fruit surface and at potential infection sites. Thus, the *in vitro* EC_50_ should be regarded as a toxicity-screening parameter rather than a direct recommendation for field application, and field trials remain necessary to determine an effective application range. Botanical and biorational products are considered promising options for reducing reliance on conventional fungicides in crop disease management systems (Fontana et al., 2021; Alexandersson et al., 2016). Increasing the formulation concentration to the highest tested level of 1,200 mg/L did not further improve field disease control. Although the *in vitro* inhibition rate continued to increase from 79.86% at 1,000 mg/L to 82.52% at 1,200 mg/L, field control efficacy decreased from 81.92% to 78.13%. This divergence suggests that the modest reduction in field efficacy at 1,200 mg/L was unlikely to result from diminished direct antifungal activity. Instead, it may be related to a less favorable physiological response of the fruit at the highest concentration. At 1,200 mg/L, the O_2_•^−^ production rate and MDA content increased slightly from their lowest levels, while the activities of SOD, POD, CAT, and PAL were generally lower than their maximum responses observed at 800–1,000 mg/L. Because allicin is a reactive sulfur-containing compound that can interact with thiol-containing proteins and permeate cellular membranes, excessive exposure may disturb cellular redox homeostasis and weaken the stimulation of defense-related responses (Rabinkov et al., 1998; Miron et al., 2000). Such effects may partly offset the additional direct antifungal activity obtained at 1,200 mg/L. Therefore, the observed response appears to reflect a concentration-dependent optimum rather than a simple linear relationship between application concentration and field efficacy, with 800–1,000 mg/L representing the most effective range under the conditions of this trial.

The control efficacy values after the second application were numerically higher than those recorded after the first application at all tested concentrations. This pattern may be explained primarily by the renewal of allicin deposits and the prolongation of protective exposure under orchard conditions. Because spray deposits on fruit surfaces may gradually decline through weathering, runoff, light exposure, and degradation or loss of active compounds, the second application performed 7 ad after the first likely restored treatment coverage on the fruit surface and fruit stalk base. Repeated application may also have suppressed newly germinating fungal propagules and protected potential infection sites that were insufficiently covered or became exposed after the first treatment. In addition, the reduced oxidative stress and enhanced defense-related enzyme activities measured after the second application suggest that repeated exposure may have helped maintain defense-related physiological responses in the fruit. However, because these physiological indicators were measured only after the second application, this possible contribution should be regarded as an interpretation rather than direct evidence of a cumulative defense-induction effect. Overall, the numerically greater efficacy after the second application likely resulted from renewed surface coverage, prolonged antifungal exposure, and possibly sustained host defense responses. However, because a formulation-only control was not included, the observed antifungal and disease-control effects should be interpreted as those of the commercial 6% allicin EC formulation under the tested conditions. Phytotoxicity and residue levels were not specifically assessed in the present study and should be evaluated in subsequent application-oriented studies.

In addition to its direct antifungal activity, allicin treatment reduced oxidative stress indicators in ‘Ancui’ hardy kiwi fruit. The O_2_•^−^ production rate and MDA content generally decreased with increasing allicin concentration and reached their lowest levels at approximately 1,000 mg/L. A slight increase was observed at 1,200 mg/L, indicating that further increasing the allicin concentration did not produce a greater reduction in oxidative stress. Excessive accumulation of reactive oxygen species can disrupt cellular redox homeostasis and cause oxidative damage, whereas MDA is widely used as an indicator of membrane lipid peroxidation and cellular membrane injury (Chen et al., 2022; Song et al., 2024). Therefore, the lower O_2_•^−^ production rate and MDA content observed in allicin-treated fruit were associated with reduced oxidative damage and better maintenance of membrane stability during disease development. These physiological changes may contribute to enhanced resistance of hardy kiwi fruit to preharvest soft rot and may help delay disease progression.

Allicin treatment also enhanced the activities of SOD, POD, CAT, and PAL in ‘Ancui’ hardy kiwi fruit. SOD and CAT are important antioxidant enzymes involved in reactive oxygen species detoxification. SOD converts superoxide radicals into hydrogen peroxide, which can subsequently be decomposed by CAT, thereby helping to maintain cellular redox balance. POD participates in antioxidant defense and may also contribute to cell wall strengthening and other defense-related processes. PAL is a key enzyme in the phenylpropanoid pathway and is associated with the biosynthesis of phenolic compounds, lignin, and other defense-related metabolites. These enzymes play important roles in antioxidant protection and induced resistance responses in plants (Hayat et al., 2016; Alexandersson et al., 2016; Su et al., 2021). The broadly similar concentration–response patterns of these enzymes, with the strongest responses occurring at approximately 800 mg/L, suggest that this concentration provided a favorable level of stimulation for the coordinated antioxidant and defense system. However, the similar optimal concentration does not imply identical regulation or response magnitude. SOD acts upstream by converting O_2_•^−^ into H_2_O_2_, whereas CAT and POD remove H_2_O_2_ through different catalytic mechanisms and substrate requirements. In contrast, PAL is a key entry enzyme of the phenylpropanoid pathway and contributes to the production of phenolic compounds, lignin, and other defense-related metabolites. Therefore, differences in basal enzyme activity, substrate availability, catalytic function, synthesis and turnover rates, and pathway-specific regulation may account for the different degrees of activation observed among SOD, POD, CAT, and PAL. Moreover, because these enzymes were measured using different activity definitions and units, their absolute activity values should not be directly compared. The common response optimum at approximately 800 mg/L may therefore reflect coordinated but functionally differentiated activation of multiple defense pathways rather than an identical response of the four enzymes. In the present study, POD, SOD, PAL, and CAT activities generally increased at intermediate allicin concentrations and declined at higher concentrations. The greatest increases in POD, SOD, and PAL activities were observed at 800 mg/L, whereas strong enzyme responses were generally maintained within the range of 800–1,000 mg/L. This concentration range was consistent with that producing the highest field control efficacy. The correspondence between enhanced defense-related enzyme activities and greater disease suppression suggests that allicin treatment may strengthen the antioxidant and defense capacity of hardy kiwi fruit. Therefore, the control of preharvest soft rot by allicin may involve not only direct inhibition of fungal growth but also enhancement of host defense-related physiological responses.

Correlation analysis further supported the relationship between disease suppression and physiological responses in ‘Ancui’ hardy kiwi fruit. Field control efficacy was negatively correlated with disease index, O_2_•^−^ production rate, and MDA content but positively correlated with SOD, POD, CAT, and PAL activities. In contrast, disease index was positively associated with O_2_•^−^ production rate and MDA content and negatively associated with defense-related enzyme activities. These coordinated relationships indicate that greater disease suppression was associated with lower disease severity and oxidative damage, together with stronger antioxidant and defense-related enzyme responses. The correlation results were consistent with the observed concentration-dependent effects of allicin. Treatments at 800–1,000 mg/L produced the greatest field control efficacy, reduced O_2_•^−^ production and MDA accumulation, and maintained relatively high defense-related enzyme activities. Although the reductions in oxidative damage and increases in defense-related enzyme activities were associated with improved disease control, the present data do not demonstrate that these responses constitute the primary mechanism of allicin-mediated disease suppression. Because pathogen colonization was not independently quantified, these physiological changes may, at least in part, represent secondary responses to reduced pathogen colonization resulting from the direct antifungal activity of allicin. Nevertheless, their coordinated concentration-dependent patterns and correlations with field control efficacy suggest that host defense-related responses may contribute to the overall disease suppression.

## 5 Conclusions

In this study, *Diaporthe* sp. isolate B4 was isolated from symptomatic fruit of the newly bred hardy kiwi cultivar ‘Ancui’ in Hunjiang District, Jilin Province, China. Morphological characterization, ITS sequence analysis, and phylogenetic analysis supported the placement of isolate B4 within the genus *Diaporthe*. Because ITS alone was insufficient for reliable species-level identification, the fungus was conservatively designated as *Diaporthe* sp. isolate B4 rather than assigned to a named species. Artificial inoculation reproduced typical soft rot symptoms, and a morphologically consistent fungus was re-isolated from the resulting lesions, fulfilling Koch’s postulates and confirming the pathogenicity of isolate B4 to ‘Ancui’ fruit. The isolate exhibited relatively broad adaptability to nutritional and environmental conditions, with the greatest mycelial growth observed under weakly acidic and relatively warm conditions.

The commercial 6% allicin EC inhibited the mycelial growth of isolate B4 in a concentration-dependent manner, with an estimated EC_50_ of 326.90 mg/L. Under field conditions, two applications of allicin at 800 and 1,000 mg/L provided the greatest control efficacy, reaching 80.09% and 81.92%, respectively. Increasing the concentration to 1,200 mg/L did not further improve disease control, indicating that 800–1,000 mg/L was the most effective concentration range under the conditions of this field trial.

Allicin treatment also reduced O_2_•^−^ production and MDA accumulation while enhancing SOD, POD, CAT, and PAL activities in fruit tissues. Field control efficacy was negatively correlated with disease index and oxidative stress indicators but positively correlated with defense-related enzyme activities. Collectively, these findings suggest that allicin-mediated suppression of preharvest soft rot may involve both direct inhibition of *Diaporthe* sp. isolate B4 and enhancement of defense-related physiological responses in hardy kiwi fruit. This study expands current knowledge of the fungal pathogens associated with preharvest soft rot of the newly bred cultivar ‘Ancui’ and provides a scientific basis for the further development of allicin as a botanical disease-management option.

## Data Availability

The ITS sequence generated in this study was deposited in GenBank under accession number PZ789839. The remaining data supporting the conclusions of this article are included in the article; further inquiries can be directed to the corresponding author.

## References

[B1] Alexandersson E. Mulugeta T. Lankinen Å. Liljeroth E. Andreasson E. (2016). Plant resistance inducers against pathogens in Solanaceae species—from molecular mechanisms to field application. Int. J. Mol. Sci. 17, 1673. doi: 10.3390/ijms17101673 27706100 PMC5085706

[B2] Chen Y. Li H. Zhang S. Du S. Wang G. Zhang J. . (2022). Analysis of the antioxidant mechanism of Tamarix ramosissima roots under NaCl stress based on physiology, transcriptomic and metabolomic. Antioxidants 11, 2362. doi: 10.3390/antiox11122362 36552570 PMC9774368

[B3] Curtis H. Noll U. Störmann J. Slusarenko A. J. (2004). Broad-spectrum activity of the volatile phytoanticipin allicin in extracts of garlic (Allium sativum L.) against plant pathogenic bacteria, fungi and oomycetes. Physiol. Mol. Plant Pathol. 65, 79–89. doi: 10.1016/j.pmpp.2004.11.00642574925

[B4] Du Y. Wang X. Guo Y. Xiao F. Peng Y. Hong N. . (2021). Biological and molecular characterization of seven Diaporthe species associated with kiwifruit shoot blight and leaf spot in China. Phytopathol. Mediterr. 60, 177–198. doi: 10.36253/phyto-12013

[B5] Fan Y. Liu K. Lu R. Gao J. Song W. Zhu H. . (2023). Cell-free supernatant of Bacillus subtilis reduces kiwifruit rot caused by Botryosphaeria dothidea through inducing oxidative stress in the pathogen. J. Fungi 9, 127. doi: 10.3390/jof9010127 36675948 PMC9862322

[B6] Fisk C. L. Silver A. M. Strik B. C. Zhao Y. (2008). Postharvest quality of hardy kiwifruit (Actinidia arguta ‘Ananasnaya’) associated with packaging and storage conditions. Postharvest Biol. Technol. 47, 338–345. doi: 10.1016/j.postharvbio.2007.07.01542574925

[B7] Fontana D. C. Dourado Neto D. Pretto M. M. Mariotto A. B. Caron B. O. Kulczynski S. M. . (2021). Using essential oils to control diseases in strawberries and peaches. Int. J. Food Microbiol. 338, 108980. doi: 10.1016/j.ijfoodmicro.2020.108980 33243629

[B8] Ge Q. Mei J. Chen S. Zhang Q. Xue Y. Yu Y. . (2020). Deposition, dissipation, and minimum effective dosage of the fungicide carbendazim in the pepper-field ecosystem. Pest Manage. Sci. 76, 907–916. doi: 10.1002/ps.5596 31441991

[B9] Han J. W. Oh M. Lee Y. J. Choi J. Choi G. J. Kim H. (2018). Crinipellins A and I, two diterpenoids from the basidiomycete fungus Crinipellis rhizomaticola, as potential natural fungicides. Molecules 23, 2377. doi: 10.3390/molecules23092377 30227680 PMC6225381

[B10] Hayat S. Cheng Z. Ahmad H. Ali M. Chen X. Wang M. (2016). Garlic, from remedy to stimulant: evaluation of antifungal potential reveals diversity in phytoalexin allicin content among garlic cultivars; allicin-containing aqueous garlic extracts trigger antioxidants in cucumber. Front. Plant Sci. 7, 1235. doi: 10.3389/fpls.2016.01235 27610111 PMC4996993

[B11] Huang L. Bian Q. Liu M. Hu Y. Chen L. Gu Y. . (2024). Structure and fungicidal activity of secondary metabolites isolated from Trichoderma hamatum b-3. J. Fungi 10, 755. doi: 10.3390/jof10110755 39590674 PMC11595493

[B12] Kim Y. S. Kim K. S. Han I. Kim M. H. Jung M. H. Park H. K. (2012). Quantitative and qualitative analysis of the antifungal activity of allicin alone and in combination with antifungal drugs. PloS One 7, e38242. doi: 10.1371/journal.pone.0038242 22679493 PMC3367977

[B13] Kobiler I. Akerman M. Huberman L. Prusky D. (2011). Integration of pre- and postharvest treatments for the control of black spot caused by Alternaria alternata in stored persimmon fruit. Postharvest Biol. Technol. 59, 166–171. doi: 10.1016/j.postharvbio.2010.08.00942574925

[B14] Larkin M. A. Blackshields G. Brown N. P. Chenna R. McGettigan P. A. McWilliam H. . (2007). Clustal W and clustal X version 2.0. Bioinformatics 23, 2947–2948. doi: 10.1093/bioinformatics/btm404 17846036

[B15] Leontiev R. Hohaus N. Jacob C. Gruhlke M. C. H. Slusarenko A. J. (2018). A comparison of the antibacterial and antifungal activities of thiosulfinate analogues of allicin. Sci. Rep. 8, 6763. doi: 10.1038/s41598-018-25154-9 29712980 PMC5928221

[B16] Li W. Long Y. Mo F. Shu R. Yin X. Wu X. . (2021). Antifungal activity and biocontrol mechanism of Fusicolla violacea J-1 against soft rot in kiwifruit caused by Alternaria alternata. J. Fungi 7, 937. doi: 10.3390/jof7110937 34829224 PMC8620048

[B17] Li Y. Shi L. B. Fei N. Y. Fu J. F. Yan X. R. (2017). Identification and biological characteristics of a blueberry Diaporthe stem canker pathogen. Plant Prot. 43, 89–94. doi: 10.3969/j.issn.0529-1542.2017.01.014

[B18] Ling L.-Z. Chen L.-L. Ma J.-Y. Li C.-Y. Zhang D.-R. Hu X.-D. . (2024). Characterization of major cell-wall-degrading enzymes secreted by Diaporthe spp. isolate Z1-1N causing postharvest fruit rot in kiwifruit in China. Biology 13, 1006. doi: 10.3390/biology13121006 39765673 PMC11673422

[B19] Lyu A. Liu H. Che H. Yang L. Zhang J. Wu M. . (2017). Reveromycins A and B from Streptomyces sp. 3–10: antifungal activity against plant pathogenic fungi *in vitro* and in a strawberry food model system. Front. Microbiol. 8, 550. doi: 10.3389/fmicb.2017.00550 28421050 PMC5376619

[B20] Lyu Z. T. (2024). “ Pathogen identification, incidence regularity and disease resistance evaluation of kiwifruit soft rot disease,” in Pathogen Identification, Incidence Regularity and Disease Resistance Evaluation of Kiwifruit Soft Rot Disease ( Anhui Agricultural University, Hefei). doi: 10.26919/d.cnki.gannu.2024.001088

[B21] Miron T. Rabinkov A. Mirelman D. Wilchek M. Weiner L. (2000). The mode of action of allicin: its ready permeability through phospholipid membranes may contribute to its biological activity. Biochim. Biophys. Acta 1463, 20–30. doi: 10.1016/S0005-2736(99)00174-1 10631291

[B22] Pereira C. Costa P. Pinheiro L. Balcão V. M. Almeida A. (2021). Kiwifruit bacterial canker: an integrative view focused on biocontrol strategies. Planta 253, 49. doi: 10.1007/s00425-020-03549-1 33502587

[B23] Portz D. Koch E. Slusarenko A. J. (2008). Effects of garlic (Allium sativum L.) juice containing allicin on Phytophthora infestans (Mont.) de Bary and on downy mildew of cucumber caused by Pseudoperonospora cubensis (Berk. & M. A. Curtis) Rostovzev. Eur. J. Plant Pathol. 122, 197–206. doi: 10.1007/s10658-008-9334-x30311153

[B24] Qiao X. Y. (2025). “ Study on the inhibitory effect of Allium sativum extract on pathogenic fungi causing Actinidia arguta rot disease,” in Study on the Inhibitory Effect of Allium Sativum Extract on Pathogenic Fungi Causing Actinidia Arguta Rot Disease ( Jiamusi University, Jiamusi). doi: 10.27168/d.cnki.gjmsu.2025.000018

[B25] Qiao X. Y. Du C. M. Li L. Li L. L. Skripchenko N. V. Liu D. (2024). Isolation and identification of pathogens of Actinidia arguta rot and antifungal study of garlic crude extract. Northern Horticulture 48, 34–42. doi: 10.11937/bfyy.20240660

[B26] Rabinkov A. Miron T. Konstantinovski L. Wilchek M. Mirelman D. Weiner L. (1998). The mode of action of allicin: trapping of radicals and interaction with thiol-containing proteins. Biochim. Biophys. Acta 1379, 233–244. doi: 10.1016/S0304-4165(97)00104-9 9528659

[B27] Ren X. J. (2007). “ Identification, biological characteristics of pathogens causing strawberry anthracnose and chemical toxicity measurements for their control,” in Identification, Biological Characteristics of Pathogens Causing Strawberry Anthracnose and Chemical Toxicity Measurements for Their Control ( Yangzhou University, Yangzhou).

[B28] Shi H. Wang R. C. Wang F. F. Wang Y. Bu F. W. Zhou Q. (2020). Isolation, identification and growth characteristics of main pathogens of kiwifruit soft rot. J. Nucl. Agric. Sci. 34, 2425–2434. doi: 10.11869/j.issn.100-8551.2020.11.2425

[B29] Song J. Xin L. Gao F. Liu H. Wang X. (2024). Effects of foliar selenium application on oxidative damage and photosynthetic properties of greenhouse tomato under drought stress. Plants 13, 302. doi: 10.3390/plants13020302 38276758 PMC10819105

[B30] Su Y. Huang Y. Dong X. Wang R. Tang M. Cai J. . (2021). Exogenous methyl jasmonate improves heat tolerance of perennial ryegrass through alteration of osmotic adjustment, antioxidant defense, and expression of jasmonic acid-responsive genes. Front. Plant Sci. 12, 664519. doi: 10.3389/fpls.2021.664519 34025701 PMC8137847

[B31] Sun X. W. Shi G. L. Ai J. Sun D. Wang Z. X. (2025). Study on genetic diversity of leaf and fruit traits of Actinidia arguta in the Changbai Mountain area. J. Jilin Agric. Univ. 48, 1–13. doi: 10.13327/j.jjlau.2025.0032

[B32] Suwannarach N. Wongsa K. Senwanna C. Nuangmek W. Kumla J. (2025). Identification, characterization, pathogenicity, and fungicide sensitivity of postharvest fungal diseases in culinary melon from Northern Thailand. J. Fungi 11, 540. doi: 10.3390/jof11070540 40985451 PMC12300269

[B33] Tamura K. Stecher G. Kumar S. (2021). MEGA11: molecular evolutionary genetics analysis version 11. Mol. Biol. Evol. 38, 3022–3027. doi: 10.1093/molbev/msab120 33892491 PMC8233496

[B34] Tan X. Tao N. (2024). Isolation and control of fruit and vegetable rot fungi. J. Fungi 10, 539. doi: 10.3390/jof10080539 39194865 PMC11355116

[B35] Wang X. Guo Y. Du Y. Yang Z. Huang X. Hong N. . (2021). Characterization of Diaporthe species associated with peach constriction canker, with two novel species from China. MycoKeys 80, 77–90. doi: 10.3897/mycokeys.80.63816 34054325 PMC8149378

[B36] Wang L. X. (2023). “ Study on the relationship between fruit development and changes of endogenous hormone content of Actinidia arguta,” in Study on the Relationship Between Fruit Development and Changes of Endogenous Hormone Content of Actinidia Arguta ( Jilin Agricultural University, Changchun).

[B37] Wu L. Q. Zhu L. W. Heng W. Ye Z. F. Liu G. Shi S. X. (2010). Identification of Dangshan pear anthracnose pathogen and screening fungicides against it. Sci. Agric. Sin. 43, 3750–3758. doi: 10.3864/j.issn.0578-1752.2010.18.00841470978

[B38] Xu Y. Li L. Zhang J. Lan Y. Li N. Wang J. (2024). Synthesis and biological activities of C1-substituted acylhydrazone β-carboline analogues as antifungal candidates. Molecules 29, 3569. doi: 10.3390/molecules29153569 39124974 PMC11314034

[B39] Yang S. Bai M. Hao G. Zhang X. Guo H. Fu B. (2021). Transcriptome survey and expression analysis reveals the adaptive mechanism of ‘Yulu Xiang’ pear in response to long-term drought stress. PloS One 16, e0246070. doi: 10.1371/journal.pone.0246070 33545712 PMC7864722

[B40] Yang C. Wu Z. Q. Qi H. Chen C. Q. Wang X. Yang L. N. . (2018). Fusarium solani, a new pathogen causing postharvest fruit rot on kiwifruit (Actinidia deliciosa) in China. Plant Dis. 102, 443. doi: 10.1094/PDIS-07-17-0939-PDN

[B41] Zhang Z. M. Hou F. M. Zhang S. Y. Li X. M. Zhang J. J. (2020). Antimicrobial and control effect of allicin on powdery mildew of Rosa roxburghii Tratt. Northern Horticulture 44, 46–51.

[B42] Zhang R. Wang S. F. Cui J. Q. Sun G. Y. (2009). Identification of pathogens causing apple fruit bitter rot in Shaanxi and Henan provinces. Sci. Agric. Sin. 42, 3224–3229. doi: 10.3864/j.issn.0578-1752.2009.09.026

[B43] Zhao J. M. Gao G. T. Gu L. J. Sun X. Y. Xue M. Geng P. F. . (2013). Identification and pharmaceutical screening of brown spot disease on Actinidia chinensis. Sci. Agric. Sin. 46, 4916–4925. doi: 10.3864/j.issn.0578-1752.2013.23.00741470978

[B44] Zhu C. (2023). “ Investigation on diseases and insect pests of citrus in Jiangshan and occurrence and management of melanose,” in Investigation on Diseases and Insect Pests of Citrus in Jiangshan and Occurrence and Management of Melanose ( Zhejiang A&F University, Hangzhou).

